# Drug Delivery Strategies for the Treatment of Pancreatic Cancer

**DOI:** 10.3390/pharmaceutics15051318

**Published:** 2023-04-22

**Authors:** Oluwabukunmi Olajubutu, Omotola D. Ogundipe, Amusa Adebayo, Simeon K. Adesina

**Affiliations:** Department of Pharmaceutical Sciences, Howard University, Washington, DC 20059, USA; oluwabukunmi.olajub@bison.howard.edu (O.O.); omotola.ogundipe@bison.howard.edu (O.D.O.); amusa.adebayo@howard.edu (A.A.)

**Keywords:** pancreatic cancer, desmoplasia, extracellular matrix, nanotechnology, drug-conjugate, immunotherapy, pancreatic adenocarcinoma

## Abstract

Pancreatic cancer is fast becoming a global menace and it is projected to be the second leading cause of cancer-related death by 2030. Pancreatic adenocarcinomas, which develop in the pancreas’ exocrine region, are the predominant type of pancreatic cancer, representing about 95% of total pancreatic tumors. The malignancy progresses asymptomatically, making early diagnosis difficult. It is characterized by excessive production of fibrotic stroma known as desmoplasia, which aids tumor growth and metastatic spread by remodeling the extracellular matrix and releasing tumor growth factors. For decades, immense efforts have been harnessed toward developing more effective drug delivery systems for pancreatic cancer treatment leveraging nanotechnology, immunotherapy, drug conjugates, and combinations of these approaches. However, despite the reported preclinical success of these approaches, no substantial progress has been made clinically and the prognosis for pancreatic cancer is worsening. This review provides insights into challenges associated with the delivery of therapeutics for pancreatic cancer treatment and discusses drug delivery strategies to minimize adverse effects associated with current chemotherapy options and to improve the efficiency of drug treatment.

## 1. Introduction

Pancreatic cancer remains one of the deadliest and most difficult cancers to treat. The disease accounts for more than 331,000 deaths per year, making it the seventh leading cause of cancer-related deaths worldwide [1,2,3,4,5,6]. Approximately 56,770 new cases of pancreatic cancer and 45,750 deaths were reported in 2019 in the United States, while 60,430 new cases and 48,220 deaths were recorded in the year 2021 [1,4,7,8]. In 2022, pancreatic cancer was reported to be the third leading cause of cancer-related death in the USA after lung and colorectal cancers with estimated new cases numbering 59,143 and deaths numbering 49,920 [9]. It is projected to be the second leading cause of cancer-related death by 2030 [1,6,10,11,12,13]. Pancreatic cancer accounts for 8% of all cancer deaths with a median survival of about six months and a meager five-year survival rate of less than 5% [1,14,15,16]. Pancreatic cancer is thus a silent killer disease that requires urgent attention.

**Figure 1:**
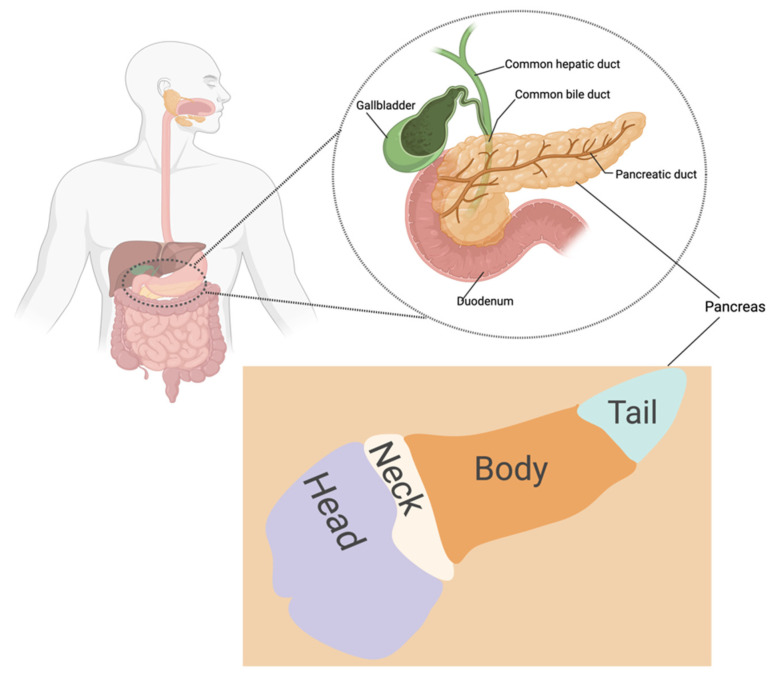
Anatomy of the pancreas and different pancreatic parts where cancer occurs. Created with BioRender.com (accessed on 14 April 2023).

Pancreatic cancer progresses slowly and asymptomatically, making early diagnosis challenging [12]. The poor prognosis of pancreatic cancer is attributed to the disease’s late diagnosis, as treatment usually commences when the tumor is at advanced stages, and to the excessive production of fibrotic stroma known as desmoplasia [11,12,14,17]. In addition, lack of efficient diagnostic techniques, early metastasis to near and distant regions, a high recurrence rate, and rigid tumor microenvironment are factors contributing to pancreatic cancer’s high mortality rate. [12,18,19]. Signs and symptoms of pancreatic cancer include nausea, stomach pain, jaundice, anorexia, weight loss, steatorrhea, and back pain and might vary from person to person depending on where the tumor is located (Figure 1) [20,21]. Risk factors include smoking, age, obesity, diabetes, pancreatitis, alcohol consumption, fatty diet, family history, and genetics [12,22,23,24,25]. Black Americans have a greater risk of developing pancreatic cancer with a higher mortality rate compared to non-Hispanic and white Americans. This may be attributed to differences in genetic makeup and socioeconomic factors [26,27].

Pancreatic ductal adenocarcinomas, which develop in the pancreas’ exocrine region, are the predominant type of pancreatic cancer, representing about 95% of total pancreatic tumors [1,14,16,19]. Neuroendocrine pancreatic cancer, which develops in the endocrine region, accounts for less than 5% of total pancreatic cancer [22,28]. It is less aggressive compared to pancreatic adenocarcinoma. Pancreatic adenocarcinoma is characterized by its dense tumor microenvironment (TME), which promotes tumor growth and metastatic spread, acts as a barrier to chemotherapy penetration, and consequently contributes to the increased rate of both primary and adaptive multi-drug resistance [5,11,18,29,30,31].

Unfortunately, despite substantial advancements in cancer therapy over the years that have extended patients’ overall life expectancies for various cancer types, there have been no appreciable changes in pancreatic cancer survival rates [1,18]. Improved clinical outcomes for pancreatic cancer require early detection and delivery of optimum therapeutic agents with minimal to no side effects on non-targeted tissues. Computed tomography or magnetic resonance imaging is usually recommended for initial examinations of suspected individuals [32,33]. Endoscopic ultrasound is also widely used in diagnosis in conjunction with other diagnostic techniques due to its ability to detect microscopic lesions [34]. However, early detection in patients does not automatically translate into reduced mortality; <35% of patients eligible for surgical intervention have a five-year rate of survival and approximately 85% of patients will experience a recurrence two years after surgery [11,16,18,34,35,36]. 

Drug delivery systems in cancer therapy refer to the various techniques and technologies utilized for conveying anticancer agents to tumor cells [37]. A broad range of drug delivery systems are employed in cancer therapy, including chemotherapy, immunotherapy, nanoparticles, drug conjugates, and combinations of these approaches [38,39]. All these delivery systems have been explored in pancreatic cancer management with the aim of improving the overall effectiveness of cancer treatment by enhancing drug efficacy and diminishing adverse effects [40].

This review provides insights into challenges associated with the delivery of therapeutics for the treatment of pancreatic cancer considering the peculiarities associated with pancreatic cancer and discusses measures to improve drug delivery efficiency.

## 2. Peculiarities of Pancreatic Cancer

The pancreas is situated in the upper abdomen, behind the stomach (Figure 1) [41]. It releases hormones and digestive enzymes that regulate the body’s metabolism and energy storage [28]. It is subdivided into four sections: the head (including the uncinate process), neck, body, and tail; pancreatic cancer can originate from any of these [20,21,41,42]. Several studies indicate that the anatomic site of pancreatic cancers influences the prognosis [20,24,43,44]. Recently, Lee and colleagues reported that head cancers had better overall survival than body/tail pancreatic cancers. The finding was attributed to the earlier onset of symptoms associated with head pancreatic tumor [24,43,44]. Patients with head cancers frequently exhibit jaundice and secondary hyperbilirubinemia due to the occlusion of the common bile duct, whereas discomfort and weight loss are typical signs of cancers of the body and tail [43].

**Figure 2:**
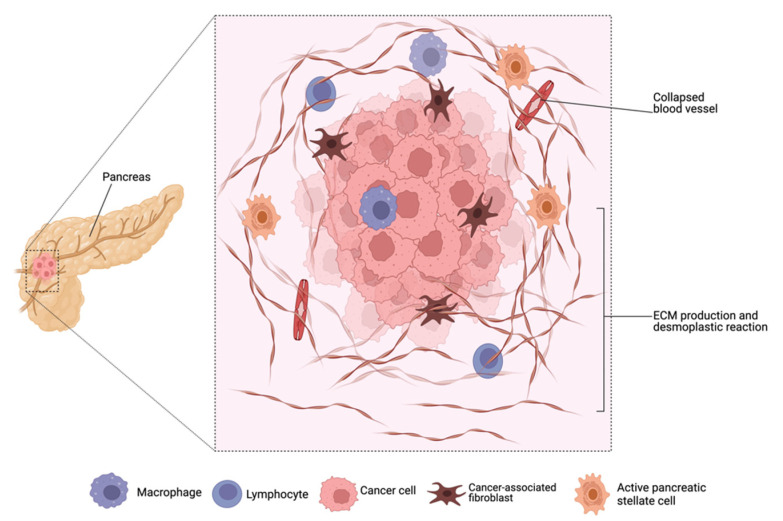
The pancreatic tumor microenvironment showing abnormal vascularity and excessive desmoplasia. Created with BioRender.com (accessed on 14 April 2023).

The initiation and progression of pancreatic cancer are multifactorial and its pathophysiology is impacted in many ways by the different components of the cancer microenvironment [45,46]. Pancreatic adenocarcinoma, unlike other malignancies, is extensively characterized by a dense fibrotic stroma, commonly referred to as desmoplasia or desmoplastic reaction (Figure 2), that aids tumor growth and metastatic spread by remodeling the extracellular matrix and releasing tumor growth factors [7,11,47,48]. Desmoplasia is established by pancreatic stellate cells and infiltrating immune cells. It acts as a barrier to chemotherapy penetration and contributes to an increase in both primary and adaptive multidrug resistance [48].

Pancreatic stellate cells (PSCs) and cancer-activated fibroblasts (CAFs) are the most prominent cellular components of pancreatic adenocarcinoma stroma [22,29,49,50]. PSCs are usually in a quiescent state in healthy individuals but become activated in pathological conditions by various mediators including cytokines, vascular endothelial growth factor (VEGF), transforming growth factor beta (TGFβ), and pancreatic parathyroid hormone-related protein [22,50]. CAFs, which secrete a variety of extracellular matrix components such as collagen, laminin, fibronectin, alpha-smooth muscle actin, fibroblast activation protein, hyaluronic acid, cytokines, tumor growth factors, and extracellular proteases, have been linked to the development of dense fibrosis in orthotopic and metastatic tumors [7,8,48,51,52,53,54]. The extracellular matrix (ECM) components secreted by CAFs have been reported to contribute significantly to pancreatic adenocarcinoma progression, making them suitable targets for therapy [46,55]. For instance, an overabundance of collagen promotes tumor rigidity, which causes blood vessel constriction and increased interstitial pressure [22,54]. As a result, the core of pancreatic tumors is hypovascularized, which elicits impaired perfusion and diffusion, thus hindering the uptake of cytotoxic agents and contributing to chemoresistance [54,56]. Depletion of stroma collagen and hyaluronic acid levels in previous studies correlate with improved overall survival [8,52,55]. Additionally, the high level of hyaluronic acid increases interstitial fluid pressure within the pancreatic tumor, which prevents penetration and uptake of cytotoxic agents [36,50]. Furthermore, stroma deposition has been correlated with increased interstitial pressure observed in the pancreatic tumor, which prevents intra-tumoral drug deposition, contributing to drug resistance [57,58]. Reports have also shown that the pancreatic adenocarcinoma stroma is rich in proteolytic enzymes, including fibroblast activation protein, matrix metalloproteinases, and transforming growth factor, which contributes to stroma remodeling [8,58,59].

Pancreatic cancer is hypothesized to start as a precancerous lesion (pancreatic intraepithelial neoplasia, PanIN) which accrues gene mutations over time, eventually developing into cancerous cells [25,49,52]. About 90% of human pancreatic cancer cells exhibit Kirsten rat sarcoma (KRAS) mutations [28,56]. KRAS plays a critical role in cell signaling pathways, and its mutation affects cancer biology in a variety of ways. It has been reported to be implicated in pancreatic cancer initiation, proliferation, apoptosis, migration, metabolism, and immune regulation [23,60]. For instance, pancreatic cancer cells thrive in hypoxic and nutrient-deficient environments because of enhanced metabolic reprogramming driven by oncogenic KRAS, which leads to increased glucose uptake, enhanced glycolysis, and lactate production in the presence of oxygen [47,61,62,63]. In addition, reduced immune cell infiltration in pancreatic cancer has been correlated with KRAS mutations [63]. Furthermore, KRAS has been considered a therapeutic target for different malignancies; however, targeting KRAS has been challenging and it has been considered “undruggable” for quite some time [22,63]. While KRAS has long been considered undruggable in several malignancies, recent advances in drug development have led to the development of new therapies that target KRAS or its downstream signaling pathways, including KRAS inhibitors, MEK inhibitors, and PI3K inhibitors, among others [63]. For example, sotorasib, a KRAS inhibitor that targets KRAS G12C mutations, was approved in 2021 by the FDA for the treatment of advanced non-small-cell lung cancer [64]. Additionally, sotorasib was recently reported as exhibiting noticeable anticancer activity in previously-treated patients with KRAS p.G12C–mutated advanced pancreatic cancer [65]. Research is ongoing to develop effective therapies for KRAS mutations in pancreatic cancer.

### 2.1. The Enhanced Permeability and Retention Effect in Pancreatic Cancer

Enhanced permeability and retention (EPR) in cancer therapy leverages the structural and functional abnormalities of solid tumors [66,67]. Blood vessels in tumors form quickly and poorly as a result of an increased rate of angiogenesis to meet the increased demands for nutrients and oxygen [68]. The newly-formed blood vessels are distorted and the endothelial cells are poorly aligned with large openings [69]. The tumor’s vascular wall also exhibits substantial structural abnormalities due to the absence of perivascular cells and smooth muscle layers [68,69]. The tumor’s leaky vasculature facilitates the transport of macromolecules and nanoparticles into the tumor tissue while the complementary poor lymphatic drainage prevents clearance of the macromolecules and nanoparticles, leading to their accumulation within the tumor tissue [51,68,70]. Thus, the differences in the blood vessels of tumors and healthy tissues make targeting possible [70,71]. This phenomenon and passive tumor targeting is referred to as the EPR effect [68,69].

Various studies have exploited the EPR effect in the delivery of macromolecular therapeutics and nanoformulations; however, the therapeutic effects vary depending on the tumor type, size, origin, and location [72,73]. Blood vessels in pancreatic adenocarcinoma may collapse or become clogged because of the dense stroma (Figure 2) and various extracellular components which induce stress and constriction, affecting the extravasation of macromolecules and particles into tumor tissues [67,74,75,76]. This may explain why nanotherapeutics have not demonstrated significant efficacy in the treatment of pancreatic cancer. However, other studies have reported that the EPR effect in cancer is not based only on the “leakiness” of the vasculature because rigid tumors such as pancreatic adenocarcinoma and prostate cancer exhibit EPR [72,73,77,78]. The combined influence of the fibrotic stroma and extracellular components such as collagen, pericytes, and melanoma fibroblasts results in impaired transport of nanoparticles into such tumors [77,79].

The hypothesis that EPR selectively promotes the accumulation of nanoparticles within tumor tissues has been greatly debated [74,80]. Recently, researchers have suggested that the increased accumulation of nanoparticles within tumors results from active transcytosis-mediated accumulation [81]. Transcytosis involves the vesicular transport of macromolecules from one side of a cell to another. Zhou et al. provided detailed insights into the transcytosis-mediated extravasation of nanomedicines [82]. Transcytosis has been reported to better facilitate the uptake of nanoparticles in a variety of “non-leaky” tumors such as pancreatic adenocarcinoma [77,81]. This is because transcytosis-mediated tumor targeting is reported to be independent of the leakiness of the tumor blood vessels when compared to the EPR effect [77,79,80]. Although transcytosis has been widely explored in the delivery of macromolecules across the blood-brain barrier into the central nervous system and in several tumors including melanoma, breast, colorectal, prostate, and ovarian cancers, the mechanism in pancreatic cancer is not fully understood and is still being investigated [77,78].

Considering the heterogeneity of the pancreatic adenocarcinoma microenvironment and the presence of dense fibrotic stroma, the EPR effect may not be sufficient for the extravasation of nanomedicines and may coexist with transcytosis [79,83]. Therefore, the development of more effective delivery systems, including the use of tumor-homing targeting peptides such as iRGD and other receptor-targeting approaches, is gaining momentum. These approaches can facilitate the uptake of anticancer agents and augment EPR effect in pancreatic adenocarcinoma [77,83].

### 2.2. Proteolytic Enzymes in Pancreatic Cancer

Proteolytic enzymes, which are also known as proteases, are believed to have important functions in cancer angiogenesis, invasion, and metastasis [84,85]. These enzymes have gained considerable attention for their essential role in cancer pathophysiology and have been extensively studied and utilized in diagnosis and drug delivery systems [84,85,86,87]. In the tumor microenvironment, proteolytic enzymes are often upregulated and several studies have utilized this phenomenon to develop strategies for targeting proteases in cancer treatment. One such approach is to use protease inhibitors that specifically block the activity of certain proteases [88]. This can prevent the breakdown of extracellular matrix components and reduce the ability of cancer cells to invade and metastasize. For an in-depth review of protease inhibitors for cancer therapy, readers are referred to the work of Rudzińska et al. [89]. Alternatively, the production of proteases in cancer cells can be targeted using small molecules or biologics that inhibit protease gene expression or the processing of protease precursors [88]. Another common approach involves the use of protease-activated prodrugs, which utilize a recognition sequence that is specifically cleaved by the target protease [90]. This design allows for the release of the active drug molecule when the prodrug encounters the target protease.

These enzymes are broadly classified into four categories: aspartate proteases, serine proteases, cysteine proteases, and metalloproteinases, based on their catalytic components [87]. In pancreatic cancer, proteolytic enzymes such as the fibroblast-activation protein (FAP), cathepsin proteases, matrix metalloproteinases (MMPs), tumor-associated trypsinogen (TAT), and uroinase-type plasminogen activator (uPA) are usually produced by the tumor cells, its dense stroma, its inflammatory cells, and its activated fibroblasts [84]. Vandooren et al. provided an insightful review detailing the involvement of proteases in cancer and drug delivery [90].

Pancreatic tumor stroma is high in cancer-activated fibroblasts, which express FAP, alpha-smooth muscle actin, and other biomarkers [91]. FAP is a type II transmembrane serine protease that belongs to the dipeptidyl peptidase IV family [92,93,94,95,96]. It is preferentially overexpressed in the tumor microenvironment of pancreatic cancer and other epithelial cancers such as ovarian, lung, prostate, and colorectal cancers and its overexpression is correlated with poor prognosis [91,92,93,95,97,98]. It is a desirable target for pancreatic cancer drug delivery because it promotes tumor cell growth and invasion through the production, deposition, and modification of the extracellular matrix [85,92,93,97,98,99]. Several groups have reported the impact of FAP in pancreatic cancer progression and its use in developing targeted drug delivery systems [91,95].

In a recent study conducted by Lin et al., anti-EGFR and anti-FAP bispecific antibody-targeted liposomal irinotecan was developed to target pancreatic cancer cells and tumor fibroblasts [100]. The formulation elicited improved cellular uptake and anticancer efficacy in a human pancreatic tumor-bearing mouse model compared to untargeted liposomal irinotecan and the results showed that fibroblast activation protein can be targeted for drug delivery. Another group of researchers developed an FAP-responsive nab-paclitaxel [2]. Overexpression of FAP in the tumor microenvironment facilitated the specific accumulation and release of the nab-paclitaxel in pancreatic cancer models.

MMPs and cathepsin proteases are another group of proteolytic enzymes extensively investigated in pancreatic cancer drug delivery [101]. MMPs are zinc-containing proteolytic enzymes that degrade extracellular matrix proteins [102]. Expression of MMP-2 and MMP-9 is associated with the growth and progression of pancreatic cancer [103,104,105]. Cathepsins have several subtypes and those most studied in pancreatic cancer are B, D, E, L, and S [17,106]. Overexpression of these enzymes is utilized in targeted drug delivery of anticancer agents. For instance, Kulkarni et al. synthesized MMP-9 cleavable PEGylated nanovesicles loaded with gemcitabine [105]. The interaction of the formulation with MMP-9 in the tumor microenvironment triggered the release of the encapsulated gemcitabine and exhibited improved anticancer activity compared to the PEGylated nanovesicles without MMP-9 substrate. Han et al. developed innovative dual-enzyme-sensitive quantum dots containing gemcitabine utilizing cathepsin B and MMP-9 cleavable linkers [107]. The formulation exhibited significant accumulation within the tumor tissue, tumor growth inhibition, and minimal toxicity compared with free gemcitabine [107]. Sulpizio et al. and Pontious et al. provided detailed reviews of the role of cathepsin enzymes in pancreatic cancer [17,108].

## 3. Recent Advances in Pancreatic Cancer Targeted Therapy and Limitations

While early diagnosis of pancreatic cancer is essential to improving patients’ survival, more effective therapies are also urgently needed [34]. Chemotherapy has been the mainstay treatment for most cancers; however, this approach has not translated to a substantial improvement in the overall clinical outcome in pancreatic adenocarcinoma patients [109].

The treatment options employed include gemcitabine plus nab-paclitaxel, capecitabine, 5-fluorouracil, and poly-chemotherapy FOLFIRINOX (folinic acid, fluorouracil, irinotecan, and oxaliplatin combination) [32]. Gemcitabine (GEM) was approved by the FDA in 1977 for the treatment of pancreatic cancer and has since been used as a monotherapy and/or in combination with other cytotoxic agents such as cisplatin, 5-fluorouracil (5-FU), and docetaxel [15,33]. GEM-based treatment is currently the go-to treatment for patients with advanced pancreatic cancer [15]. However, reports have shown that pancreatic adenocarcinoma remains refractory to conventional chemotherapeutics primarily because of the disease’s genetic heterogeneity and dense stroma, which inhibits penetration and accumulation of chemotherapeutics at the tumor sites. Hence, to improve the efficiency of pancreatic cancer therapy, selective penetration and accumulation of cytotoxic agents must be enhanced [19,35,110].

Chemotherapeutic agents lack the ability to differentiate between cancer cells and healthy cells, increasing the risk of unwanted toxicity [15,39,68,111]. Additionally, some of these cytotoxic agents exhibit poor bioavailability resulting from biological degradation, physiological barriers, and low tumor penetration, all of which account for low tumor–drug concentration and therapeutic failure. Different targeting approaches have been employed to offset the limitations of chemotherapy, including nanotherapeutics, immunotherapy, and several combination approaches [38,39]. These are highlighted below.

### 3.1. Immunotherapy Approaches for the Treatment of Pancreatic Cancer

**Figure 3:**
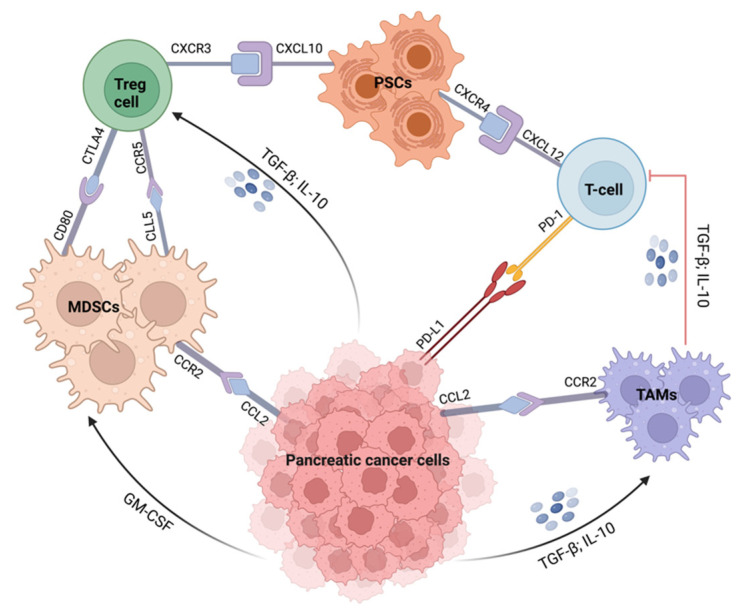
Representation of cross-interaction of different cells in the pancreatic tumor microenvironment (TME). The cancer cells, through cell surface molecules and soluble cytokines such as transforming growth factor (TGF-β) and interleukin (IL-10), establish immunosuppressive TME by recruiting and activating immunosuppressive cells such as regulatory T cells (Tregs), myeloid-derived suppressor cells (MDSCs), and tumor-associated macrophages (TAMs). In addition, interaction between programmed cell death protein ligand-1 (PD-L1) on the cancer cells and programmed cell death protein-1 (PD-1) expressed on T cells induces T cell apoptosis, resulting in immune system evasion. These immunosuppressive cells and their associated molecules are targets for cancer immunotherapy. Created with BioRender.com. Accessed on 14 April 2023. Adapted from [113].

The use of immunotherapy, which involves the activation of the immune system against cancer progression, has gained popularity [112]. Immunotherapy aims to target and alter the activation of the tumor microenvironment (TME) stromal and immunosuppressive cells, such as regulatory T cells (Tregs), myeloid-derived suppressor cells (MDSCs), and tumor-associated macrophages (TAMs), as well as the secretion of cytokines and other immune cells to cancer sites (Figure 3) [59,62,113,114]. Immunotherapies that are being exploited in pancreatic cancer treatment include immune checkpoint inhibitors, adoptive T-cell therapy, specific immunomodulators, vaccines, and combinations of these immunotherapeutic agents [60]. Immune checkpoint blockade regulates T-cell activation and induces cancer death by inhibiting cytotoxic T-lymphocyte-associated antigen-4 (CTLA-4) and programmed cell death protein-1 (PD1) ligands [114,115,116].

CTLA-4 competes with CD28 to bind to B7-1 (CD80) or B7-2 (CD86) ligands on the surface of activated T cells, inhibiting CD28-mediated stimulatory signal which leads to the downregulation of T cell function and immune suppression [116,117]. Blockades of CTLA-4 in several cancers including melanoma, renal cell carcinoma, and colorectal cancer have demonstrated remarkable efficacy [118]. Ipilimumab, a fully humanized antibody, is a CTLA-4 inhibitor that promotes antitumor activity by inhibiting CTLA-4 interaction with B7-1/B7-2 to enable T cell activation. The FDA approved ipilimumab in 2011 for unresectable or advanced metastatic (stage III or IV) melanoma. In pancreatic cancer treatment, the use of ipilimumab alone was not effective and its combination with other immunotherapeutics and chemotherapy agents has been suggested. In a Phase 1b clinical trial by Kamath et al., combining ipilimumab with gemcitabine did not demonstrate improved anticancer effects compared with gemcitabine alone [119]. In another study conducted by Wu et al., the anticancer effect of ipilimumab and GVAX vaccine was evaluated in metastatic pancreatic cancer using FOLFIRINOX as standard treatment [120]. The combination did not demonstrate improved overall survival over chemotherapy and the treatment failure was related to the tumor’s counterregulatory pathways, which prevented the induction of potent anticancer effects in pancreatic cancer [120].

PD1 antagonists including pembrolizumab, nivolumab, and durvalumab are being used in the treatment of some malignancies. Programmed cell death protein ligand-1 (PD-L1) is overexpressed in pancreatic cancer and its presence has been associated with poor prognosis [121]. Nivolumab and pembrolizumab were approved by the FDA in 2014 for the treatment of melanoma after demonstrating significant progression-free survival compared to chemotherapy [116,122]. PD1 antagonists have not demonstrated improved clinical outcomes in pancreatic cancer therapy, even in combination with chemotherapeutic agents. Weiss and colleagues conducted a phase Ib/II study (NCT02331251) of chemotherapy plus immunotherapy combination using gemcitabine, nab-paclitaxel, and pembrolizumab in 17 histologically-confirmed pancreatic adenocarcinoma patients [123]. The study’s primary goal of >15% complete response was not achieved, with a meager 9.1- and 15-month progression-free survival and overall survival rate, respectively. In addition, in a recent phase I study of nivolumab combined with nab-paclitaxel plus gemcitabine in advanced pancreatic cancer conducted by Wainberg et al., the combination therapy did not improve patients’ overall survival [124]. The clinical outcomes do not support further study [124].

Therapeutic vaccines which stimulate the host’s immune system to develop anti-tumor immunity to fight cancer cells have also been explored in pancreatic cancer. These include antigen-specific and whole-cell vaccines. Several trials are investigating the effectiveness of cancer vaccines and their combination with chemotherapy and immune checkpoint inhibitors; however, only a very few have demonstrated improved efficacy [112,116]. The combination of cyclophosphamide and GVAX, an irradiated allogeneic whole-tumor cell vaccine with CRS-207, a live–attenuated mesothelin-expressing Listeria monocytogenes vaccine, demonstrated no improvement in overall survival compared with the chemotherapy-only group [125]. Likewise, Middleton and colleagues investigated the antitumor effect of the combination of gemcitabine and capecitabine with or without GV1001, a telomerase peptide vaccine, in phase III clinical trials [126]. The findings showed that the combination of GV1001 with chemotherapy did not improve overall survival when compared with the chemotherapy alone group (7.9 months vs. 6.9 months, respectively).

Immense efforts have been harnessed toward developing more effective immunotherapeutics for pancreatic cancer treatment. Although these approaches have the potential to induce robust antitumor immunity, they have so far failed to deliver on their preclinical potential in pancreatic cancer [60,118]. Pancreatic TME is highly immunosuppressive and considered to be unfavorable for immunotherapies [118,127]. In addition, the lack of improved efficacy has been attributed to the presence of dense stroma and extracellular matrix components in pancreatic cancer, which prevents the uptake of cytotoxic agents by acting as a biophysical barrier [55,116,126]. For in-depth studies on immunotherapy approaches for the treatment of pancreatic cancer, the reader is referred to excellent reviews by Torphy et al., Schizas et al., and Di Federico et al. [118,127,128].

### 3.2. Photodynamic Therapy in Pancreatic Cancer

Another promising treatment approach that is gaining attention in pancreatic cancer treatment is photodynamic therapy (PDT). PDT is a non-invasive cancer treatment approach that uses photosensitizing agents and light of specific wavelengths to trigger localized tissue necrosis [68,129,130,131,132,133]. Typically, the photosensitizer is administered orally or intravenously and has a tendency to selectively accumulate in abnormal or cancerous cells. The use of macromolecular photosensitizers in PDT aids in the preferential accumulation of the photosensitizer in neoplastic tissues and inhibits rapid clearance from those tissues [129]. Following exposure to the specific light wavelength, the photosensitizer becomes activated and transmits absorbed photon energy or excited electrons to neighboring oxygen molecules, generating reactive oxygen species (ROS), specifically singlet oxygen species, that cause damage to nucleic acids and proteins, subsequently leading to cancer cell death [68,132]. The therapeutic light can be administered to the intended area directly, through endoscopy, or via a customized probe [134]. PDT has found application in the treatment of various cancers including brain, skin, breast, lung, and pancreatic cancers. The treatment strategy has been reported to possess several advantages including minimal invasiveness, flexibility and tissue selectivity over conventional treatment approaches. [130,133,135]. 

In pancreatic cancer, PDT has been explored as an adjuvant in combination with chemotherapy, radiotherapy, immunotherapy, and surgical resection [130]. Different photosensitizers including verteporfin, mesotetrahydroxyphenyl chlorin, photofrin, and sodium porfimer have been evaluated in the treatment of pancreatic cancer [132,133]. Verteporfin-based PDT has been shown to exhibit good cytotoxic effects in some gemcitabine-resistant pancreatic cancer cells [136]. Lu et al. reported the efficacy of verteporfin and sodium porfimer in different pancreatic cell lines [132]. Their results indicated that both photosensitizers induced dose-dependent cell death with varying sensitivity to different cell lines, but verteporfin had a greater efficacy at a much lower concentration compared with sodium porfimer. Huggett et al. in their phase I/II study reported on the efficacy and safety of vertepofin in inducing tumor necrosis in locally-advanced pancreatic cancer [131]. Additionally, Xie et al. investigated the antitumor effect of combination chemotherapeutic and PDT agents, gemcitabine, and photosan respectively [130]. Their findings showed that PDT elicited significant anticancer activity for a short duration. Based on the results, combining chemotherapy and photodynamic therapy is recommended for enhanced anticancer activity [130,136,137].

PDT is an effective treatment for cancer, but its effectiveness depends on various factors such as the choice of photosensitizer, optimal dosage, and penetration depth of the light. The intracellular uptake and localization of the photosensitizers, as well as vascular permeability, play a crucial role in the selective accumulation of drugs and cytotoxic response [132]. The clinical application of PDT in treating pancreatic cancer has been limited by factors such as the rigid fibrotic stroma surrounding pancreatic tumors limiting effective delivery of photosensitizers, dependence on tumor oxygenation, and imprecise dosimetry [134,138,139]. The reader is referred to review by Wang et al. for details on PDT of pancreatic cancer [133].

**Figure 4:**
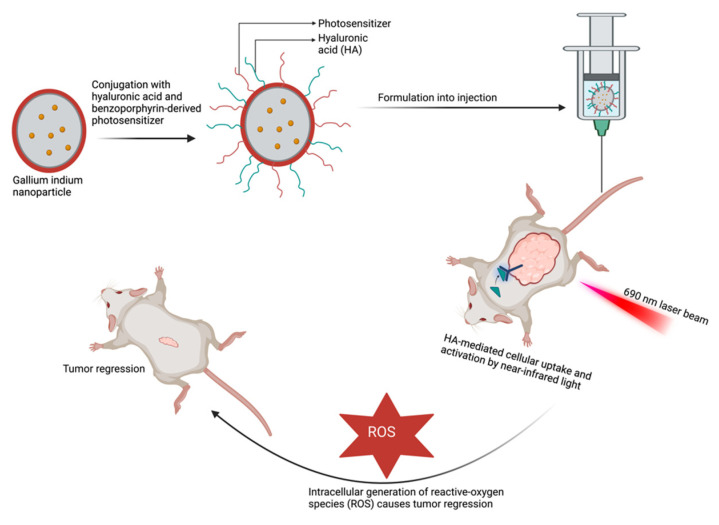
Photodynamic therapy using gallium–indium nanoparticles that have been modified with hyaluronic acid (targeting agent) and a benzoporphyrin derivative photosensitizer. Created with BioRender.com (accessed on 14 April 2023). Adapted from [139].

To improve the clinical application of PDT, delivery systems utilizing conjugated polymers and the encapsulation of photosensitizers within nanovehicles are being explored [135]. For instance, conjugation of pheophorbide-a, a PDT agent, with nanoparticles improved the efficiency of pheophorbide-a and the overall efficacy of the fabricated delivery system [137]. Using nanotechnology and conjugate delivery systems in PDT provides additional benefits such as enhancing targeting and selectivity. These delivery systems can be further functionalized by incorporating tumor-targeting and cell-penetrating molecules, improving the precision of the treatment. A good example is the novel drug-delivery system developed by Hafiz et al. using a liquid metal nanoplatform (Figure 4) [139] This formulation consists of eutectic gallium–indium nanoparticles conjugated with hyaluronic acid and benzoporphyrin derivative serving as a targeting ligand and photosensitizer, respectively. The system exhibited significant cellular uptake and tumor targeting and, upon activation by near-infrared light, markedly increased the intracellular ROS, leading to tumor regression and higher necrosis compared with the control group.

Managing pancreatic cancer has been extremely difficult and it requires a multimodal approach for optimal therapy. Therefore, exploring PDT as an adjuvant therapy should be further investigated. For further readings on photodynamic therapy, the reader is referred to the excellent reviews by Meng et al. [135].

### 3.3. Nanootechnology in the Treatment of Pancreatic Cancer

Nanotechnology is a multifaceted branch of science that entails the manufacturing of materials and devices at the nanometer scale [140]. Nanotechnology is widely employed in medicine and is one of the most popular approaches in cancer-targeting drug design and diagnosis [31,140]. Nanotechnology has been greatly studied in cancer research for improved delivery of anticancer agents utilizing the leaky vasculature of the tumor via EPR-based passive targeting and/or active targeting [3,15,141]. Recent research focuses more on active targeting and/or a combination of both passive and active targeting for a wider range of applications in enhancing drug delivery efficiency and minimizing off-target toxicity compared with passive targeting only [141,142]. Additionally, some tumors do not exhibit the enhanced permeability effect and the vascular permeability within a tumor and between tumor types varies [36,69,75,142]. Tumors can be targeted actively by employing a variety of strategies that allow selective delivery of anticancer agents to the target region.

**Figure 5:**
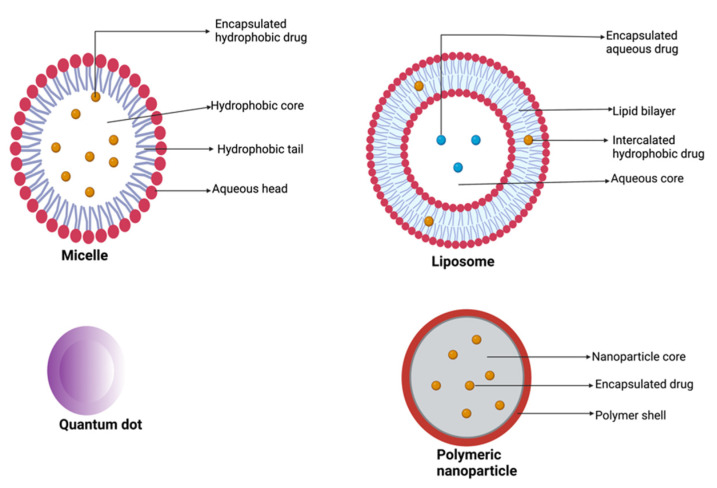
Nanoparticulate drug delivery systems. Created with BioRender.com (accessed on 14 April 2023).

Nanoparticulate drug delivery systems involve the use of a wide variety of nanocarriers including liposomes, polymeric nanoparticles, micelles, gold nanoparticles, and quantum dots (Figure 5) [3,31,143]. These nanocarriers have been exploited extensively and several nano-products have been fabricated as improvements over conventional chemotherapeutics. In the design of nanoproducts, therapeutic agents can be conjugated to or encapsulated within the nanocarriers [36]. Nanoparticle-based drug delivery systems are designed to ensure effective and efficient delivery of cytotoxic agents to the tumor site at high concentrations compared with free drugs, improve the therapeutic agent’s pharmacokinetic profile factors such as solubility, half-life, and mean residence time, and minimize off-target exposure and toxicities [31,144,145]. Conversely, the high cost of production, burst release of the encapsulated drug, poor stability in the systemic circulation, off-target tissue accumulation, large molecular size, nanotoxicity, and batch-to-batch variation are some of the limitations associated with the use of nanovectors in drug delivery [144]. Burst or rapid release of large proportions of drugs loaded in nanocarriers within a short period following administration before reaching the target site is one of the main limitations that has affected the clinical translation of some of the nanoformulations [141]. This phenomenon can lead to therapeutic failure and severe toxicity [146,147].

The development of stealth nanoparticles, also known as PEGylated nanoparticles, is an important approach utilized to alleviate some of the limitations of nanoparticulate drug delivery systems, such as instability in circulation, short half-life, and rapid clearance [148]. Polymers such as polyethylene glycol (PEG) and N-(2-hydroxypropyl)methacrylamide (HPMA) copolymers are adsorbed or covalently bonded to nanoparticle surfaces to provide a steric barrier [68,148,149,150]. This approach confers prolonged nanoparticle circulation via reducing systemic clearance by minimizing reticuloendothelial system uptake and improves the pharmacokinetic profile of the encapsulated active pharmaceutical ingredients leading to reduced toxicity and enhanced therapeutic efficacy [150].

#### 3.3.1. Albumin-Based Nanoparticles for the Treatment of Pancreatic Cancer

Albumin is a protein nanomaterial from different natural sources used as carriers for a wide variety of compounds. Albumin constitutes the highest fraction of human plasma protein and is commonly used in the synthesis of albumin-based nanoparticles [151,152]. Albumin-based nanoparticles are popular in cancer therapy and are one of the most extensively studied nanocarrier systems in the treatment of pancreatic cancer clinical trials [33,62,145]. They are biocompatible, biodegradable, and well-tolerated with a high safety profile. Albumin nanocarriers have been reported to improve the stability of various therapeutic payloads, enhance drug uptake and subsequent accumulation within tumors, and facilitate prolonged circulation time [137,152,153,154,155,156]. On the other hand, because albumin is an endogenous protein, there may be batch-to-batch variations in size and purity of albumin-based products, which may affect commercial scalability. Additionally, the organic solvents utilized in manufacturing processes may predispose albumin to denaturation and unwanted reactions. Furthermore, albumin may react with other endogenous components in the body, increasing the risk of immunogenicity and instability during systemic circulation [152,157]. Bovine serum albumin (BSA) is a commonly-used alternative to human serum albumin. BSA is relatively cheap, structurally similar to human serum albumin, and elicits negligible immunogenicity [152,158]. Detailed reviews on albumin and its uses as a nanocarrier are available elsewhere [151,159,160].

Abraxane^®^ was the first albumin-based nanoparticle to be approved in 2013 [156,161]. Abraxane^®^, also known as nab-paclitaxel (nanoparticle albumin-bound paclitaxel), is an albumin-stabilized paclitaxel authorized for first-line treatment of metastatic pancreatic cancer in combination with gemcitabine. The nanoformulation demonstrated improved overall survival and notable safety profile compared to the conventional use of the chemotherapeutic agent [158]. Additionally, in a phase III clinical trial conducted by Goldstein and colleagues in 2015, the study reported the efficacy of nab-paclitaxel and gemcitabine in combination over gemcitabine alone with overall survival of 8.7 months in the combination group versus 6.6 months in the gemcitabine group, with a median difference of 2.1 months [161].

Several other albumin-based formulations have been investigated in pancreatic cancer treatment and bioimaging to improve therapeutic effect and minimize side effects [137,152,155,156,158]. Albumin nanoparticles co-loaded with paclitaxel and curcumin demonstrated enhanced anti-tumor activity in vivo and in vitro and a controlled-release effect [153]. Albumin as a nanocarrier is considered effective for loading multiple drugs, is versatile, and can be effectively functionalized [162]. Another innovative delivery system evaluated for pancreatic cancer therapy is the enzyme-sensitive, albumin-based gemcitabine theranostic [163]. The formulation was fabricated by conjugating gemcitabine to an albumin nanocarrier via a cathepsin B cleavable linker, then complexing with IR780. IR780 is a near-infrared dye used in cancer imaging and phototherapy. Direct use of IR780 in cancer therapy is discouraged because of associated toxicity [164]. The albumin-based design resulted in a prolonged retention effect of IR780 compared to free IR780 and significantly increased gemcitabine concentrations in tumor tissue and with minimal unwanted toxicity [163]. Similarly, a multi-functional albumin-based nanoparticle was synthesized by Yu and coworkers for the delivery of gemcitabine and a photodynamic agent, pheophorbide, in pancreatic cancer with lymph metastasis [137]. Gemcitabine’s short half contributes to its low tumor tissue concentration and eventual therapeutic failure. Also, pheophorbide is hydrophobic and its free administration impaired its photodynamic effect. The albumin-based formulation improved individual shortcomings of gemcitabine and pheophorbide. Data from the study showed that the delivery system effectively inhibited the growth of both primary and metastatic tumors and offered efficient imaging-guided drug delivery [137]. However, despite extensive research on the use of albumin in drug development, only a few albumin-based therapies have advanced to the clinical stage.

#### 3.3.2. Liposomal Nanoformulations for Pancreatic Cancer Treatment

Another nanocarrier commonly used in the delivery of anticancer agents is liposomes [36]. Compared with other nanoparticles, liposomes have demonstrated great success, accounting for more than 60% of all approved nanoproducts [165,166,167]. Liposomes are bi-layered phospholipid vesicles with a hydrophilic core. Liposomes are suitable drug carriers for the delivery of hydrophilic and hydrophobic payloads [143]. They are considered promising because of their excellent biocompatibility, biodegradability, small size, and low toxicity profiles [165,167,168]. Liposomal drug delivery systems have been reported for site-specific delivery because of their tendency to accumulate in tumor tissues via the enhanced permeability and retention effect and they are also suitable for surface functionalization in active targeting [169].

Various liposome-based formulations have been developed and evaluated in the treatment of pancreatic cancer. Ranjan and colleagues synthesized liposomal curcumin and established its anticancer effect against a pancreatic cancer xenograft model. Curcumin is an anticancer agent of natural origin, but its hydrophobicity and low systemic bioavailability reduce its efficacy. The study showed that liposomal curcumin exhibited a significant anticancer effect compared with free curcumin [170].

Zinger and co-researchers developed collagozome, a collagenase nanoliposomal formulation aimed to improve the penetration of paclitaxel micelles in pancreatic adenocarcinoma [171]. Overexpression of collagen confers rigidity and contributes significantly to the development of a dense stroma. Collagozome contains collagenase enzymes that degrade collagen and reduce fibrotic tissue in pancreatic adenocarcinoma. The liposomal formulation demonstrated a prolonged collagenase release rate and protected collagenase from early degradation in the plasma [171]. The overall efficacy was attributed to the effectiveness of liposomal collagozome in remodeling the tumor extracellular matrix and the approach has been recommended for future work. Another recent development in the liposomal delivery system is the co-delivery of gemcitabine and Mcl-1 siRNA using liposomes as the nanocarrier (LPGem-siMcl-1). Mcl-1 siRNA is a type of small interfering RNA (siRNA) molecule that is designed to target and silence the expression of the myeloid cell leukemia-1 (Mcl-1) gene leading to an increase in apoptosis and a potential decrease in cancer cell growth. Mcl-1 is an anti-apoptotic protein that plays a critical role in regulating programmed cell death (apoptosis) in a variety of cell types, including cancer cells. The liposomal system was reported to effectively deliver the two active agents into the pancreatic tumor, protected the agents from early degradation, and elicited improved anti-tumor activity [172]. However, considering the average particle size (188.7 nm) of the liposomal formulation and the reported dependence of therapeutic efficacy on particle size, the effective delivery in the pancreatic adenocarcinoma model is worthy of further study to ascertain the mechanism of the reported activity. In another report, Ji et al. synthesized β-cyclodextrin (β-CD) modified MMP-2 responsive liposomes containing gemcitabine, a chemotherapeutic agent and pirfenidone, an antifibrotic agent [167]. Pirfenidone was incorporated in the β-CD, and gemcitabine was encapsulated in the liposomes. β-CDs were ligated to the liposome with an MMP-2 cleavable peptide. The liposome was also modified with Arg-Gly-Asp (RGD) peptides for targeting tumor cells. Pirfenidone was first released from the formulation following exposure to MMP-2 in the tumor microenvironment, decreasing the dense fibrotic tissue and promoting subsequent uptake and accumulation of the gemcitabine-loaded liposomal product compared with free gemcitabine.

While liposomes have been extensively studied for more than five decades, Onivyde, a liposomal irinotecan injection, is the only liposomal product approved by the FDA in 2015 for the treatment of metastatic pancreatic cancer [165,168,169]. Onivyde is used as a second-line treatment option in combination with chemotherapy for metastatic pancreatic cancer following gemcitabine-based therapy [173]. In the global phase 3 clinical trial conducted by Wang-Gillam et al., Onivyde in combination with 5-fluorouracil and leucovorin (5-FU/LV) demonstrated improved overall survival of 6.1 months versus 4.2 months in patients who received 5-FU/LV alone [174]. The median progression-free survival for the combination of nanoliposomal irinotecan and 5-FU/LV was 3.1 months compared to 1.5 months in 5-FU/LV alone [174]. The meager 6.1 months of overall survival is an indication that the prognosis of pancreatic cancer is still poor and that more potent treatment is clearly needed.

Liposomes are versatile in drug delivery and much work is still being done to develop multifunctional liposomes. Multifunctional liposomal drug delivery systems are designed to elicit multiple functions. They can be engineered to respond to specific stimuli, such as changes in pH, temperature, or enzyme activity which can trigger the release of the encapsulated drug at the desired site of action. They may also be modified by targeting ligands such as monoclonal antibodies, proteins, aptamers, and peptides, to minimize unwanted effects of anticancer agents on healthy cells and increase selective delivery to tumor cells. These multifunctional liposomes have demonstrated preclinical success [168,175]. Although these nanoformulations have been reported to demonstrate improved pharmacokinetic profiles and therapeutic effects when compared with the active agent in preclinical findings, they have not been translated clinically [168]. Several liposomal formulations are at preclinical stages and in clinical trials and it is expected that more novel liposomal formulations with improved efficacy will be available in the near future [165,166,168].

#### 3.3.3. Polymeric Nanoparticles for the Treatment of Pancreatic Adenocarcinoma

Polymeric materials are widely used as drug delivery carriers and allow for conjugation and encapsulation of chemotherapeutic and immunotherapeutic agents. They have excellent biocompatibility and can be functionalized for targeted drug delivery [176]. Conjugation or encapsulation of cytotoxic agents in polymeric nanoparticles enhances the delivery of poorly-soluble drugs, prolongs half-life, and enhances drug accumulation at the tumor site. Different polymeric nanocarriers have been investigated for the treatment of pancreatic cancer including polymeric micelles, dendrimers, nanogels, and polymeric nanoparticles. Polymeric nanoparticles are made from several polymers such as poly-(lactic-co-glycolic acid) (PLGA), polyglycolic acid (PGA), polyamidoamine (PAMAM), and polylactic acid, etc. [36,143,177,178].

In a study conducted by Wu et al., PEGylated PLGA nanoparticles were loaded with paclitaxel and the surface was functionalized with tumor-specific mucin-1 antibody (TAB004) [179]. Mucin-1 is overexpressed in more than 80% of pancreatic adenocarcinomas and is associated with increased metastasis and poor prognosis. PEG-PLGA was a suitable nanocarrier with high loading efficiency and PEGylation confers prolonged circulation and reduced systemic clearance. The modest cellular internalization and accumulation exhibited in an in vitro study were attributed to the reaction between the conjugated antibody and antigen expressed by the tumor cells [179]. Further, Sun and colleagues also developed a small particle-size (15.40 nm) redox-responsive gemcitabine polymer co-loaded with paclitaxel and an immunomodulating agent, NLG919 [180]. The resulting micelles elicited significant anticancer activity in the pancreatic (PANC02) xenograft model. The increased anti-tumor activity was attributed to the size of the micelles which further supports the notion that uptake of large particle-size nanoproducts is hindered by the dense stroma in pancreatic cancer. Additionally, the study demonstrated the synergistic effect of the co-delivery of chemotherapeutic agents and immunotherapeutics [180].

To optimize the effectiveness of nanodrugs, attention has recently been focused on the use of smart nanocarriers, which allow for surface functionalization for improved selectivity in targeted drug delivery [181]. These nanocarriers are capable of site-specific drug uptake and drug release upon stimulation by physiological or external stimuli. However, despite the tremendous effort of researchers positioning nanotechnology as the solution to the limitations of chemotherapeutics, the gap between the pre-clinical findings and clinical trial results is still very wide [36,145]. As promising as nanoparticle delivery systems seem, only a few have been used clinically for the treatment of pancreatic cancer. We identified three novel nanoparticle systems that are currently in clinical trials for pancreatic cancer therapy (Table 1). Imx-110 is a water-soluble, nano-sized formulation composed of nanoparticles encapsulating the poorly water-soluble curcumin and the antineoplastic anthracycline antibiotic, doxorubicin. Curcumin has demonstrated anticancer activities in different types of cancer, including pancreatic cancer, by targeting multiple signaling proteins such as the signal transducer and activator of transcription 3 (STAT3) and nuclear factor Kappa B (NF-kB) [182,183]. These proteins are involved in cancer initiation and resistance to chemo-radiation therapies and targeted agents [182]. Delivery of doxorubicin in Imx-110 nanoparticles may improve drug penetration into tumors. Additionally, co-delivery with curcumin, by inhibiting NF-kB and STAT3 activity, may circumvent the tumor cells’ multidrug resistance mechanisms and may therefore be effective in chemoresistant tumor cells. NBTXR3 is a 50 nm nanoparticle containing inert inorganic hafnium oxide (HfO2) crystals that has shown clinical activity in hepatocellular carcinoma and advanced solid malignancies with lung or liver metastases [184]. Subsequent application of radiation after intratumoral injection of NBTXR3 triggers the activation of NBTXR3 at the target site, which in turn causes targeted destruction of the cancer cells by enhanced absorption of ionizing radiation [184]. Because NBTXR3 is inert, it only emits electrons when exposed to radiation, thereby increasing the effectiveness of radiotherapy in comparison to conventional radiotherapy alone. Lastly, AGuIX-NP is a 4 ± 2 nm hydrodynamic diameter polysiloxane-based nanoparticle that contains gadolinium, a paramagnetic contrast enhancer for theranostic purposes [185]. AGuIX-NP can passively accumulate in tumor microenvironments due to the EPR effect following intravenous administration. Its small size also enables deep penetration into the tumor, as well as rapid renal clearance.

## 4. Drug-Conjugate Delivery Systems in Pancreatic Cancer Treatment

**Figure 6:**
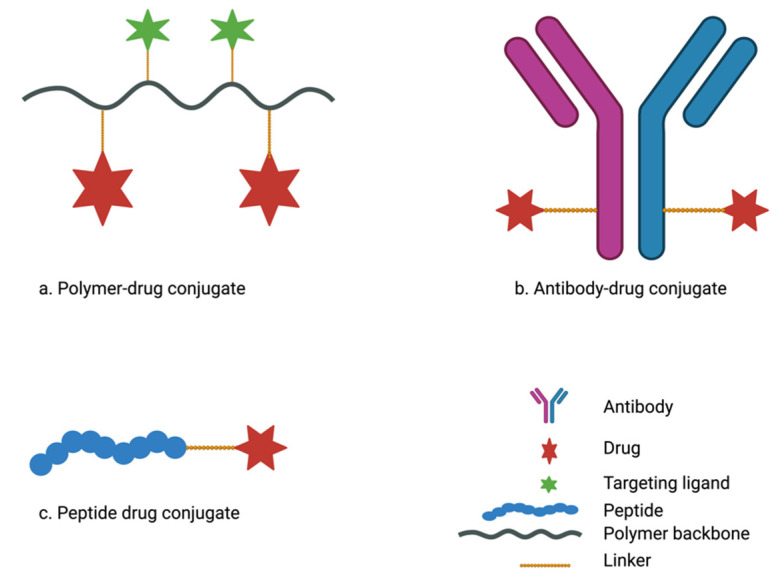
Different types of drug conjugates that are used in cancer therapy. Created with BioRender.com (accessed on 24 February 2023).

To enhance drug delivery efficiency to desmoplastic pancreatic adenocarcinoma, drug conjugates are gaining popularity owing to their smaller size and the possibility of achieving specific tumor targeting [186]. Targeting tumor-specific components or overexpressed receptors on cancer cell surfaces with monoclonal antibodies and peptides could be a positive approach to address challenges in pancreatic cancer treatment. Drug conjugate systems, including polymer-drug conjugates, antibody-drug conjugates, and peptide-drug conjugates (Figure 6), have demonstrated advantages in cancer treatment and are seemingly promising for pancreatic adenocarcinoma treatment compared with conventional nanoparticulate drug delivery systems [187,188]. Drug conjugates use the “pro-drug” approach to covalently link drug molecules with macromolecules via suitable linkers [189]. The “pro-drug” strategy modifies the drug’s physicochemical and pharmacokinetic profiles and conceals bioactivity as it remains inactive in circulation until it arrives at the target sites. Drug conjugates as delivery systems are easily synthesized, are more versatile, and minimize off-target toxicity of chemotherapeutic agents compared with nanoparticle drug delivery systems [189,190].

### 4.1. Polymer-Drug Conjugates for the Treatment of Pancreatic Cancer

A number of polymer–drug conjugates, commonly referred to as polymeric prodrugs, have generated significant interest and are presently being evaluated in clinical studies for the treatment of various disease conditions [191]. Generally, the polymer–drug conjugate is made up of a hydrophilic polymer and cytotoxic agents which are covalently linked directly or via suitable stimuli-responsive linkers (Figure 6a) such as peptide linkers GFLG (glycyl-phenylalanyl-leucyl-glycine) and PLGLAG (Pro-Leu-Gly-leu-Ala-Gly) [192,193]. Polymer–drug conjugates, upon exposure to stimuli such as relatively lower pH or enzymes at the tumor site, undergo cleavage of substrate linkers between the polymer backbone and the drugs leading to the release of cytotoxic agents [193]. Thus, polymer–drug conjugates facilitate site-specific drug delivery and reduce premature or off-target drug release and unwanted toxicity to healthy cells, which are common phenomena in nanoparticulate delivery systems [191,193]. Polymer–drug conjugates confer increased aqueous solubility, prolonged drug circulation, and enhance drug accumulation within the tumor microenvironment via the EPR effect. Compared with the conventional polymeric nanoparticulate system, which involves encapsulation of the therapeutic drug within polymeric nanocarriers, the polymeric-drug conjugate system exhibits a higher loading capacity and improved controlled drug release [193].

Polyethylene glycol-betulinic acid (PEG-BA) is a novel simple polymer–drug conjugate developed by Mosiane et al. [194]. Betulinic acid is a potent anti-cancer agent of medicinal plant origin; however, its direct use is discouraged because of its poor solubility, short half-life, and high molecular weight, which impedes cellular uptake. PEG-BA exhibited enhanced anticancer and antioxidant effects compared with free betulinic acid [194]. Thus, it is shown that the conjugation of betulinic acid to polyethylene glycol confers an improved pharmacokinetic profile and pharmacological activity on the compound.

Wang et al. synthesized a polyamidoamine dendrimer-camptothecin conjugate for the treatment of pancreatic cancer [133]. In this work, camptothecin was covalently attached to the dendrimer via a reactive oxygen species (ROS)-sensitive thioketal linker and surface modified with glutathione. Gamma(γ)-glutamyl transpeptidase (GGT), which is extensively expressed on the membrane of pancreatic cancer, triggered the conversion of glutathione to amines via a charge-reversal mechanism by catalyzing γ-glutamyl transfer reactions of glutathione to generate primary amines which confer a positive charge on the dendrimer-camptothecin conjugate. Glutathione is negatively charged at neutral pH and the switch to positive charge facilitates penetration and uptake of the conjugate via caveolae-mediated endocytosis and transcytosis allowing the deep penetration into the tumor. It has been reported that to achieve efficient delivery of nanoparticles to tumors, a surface charge that is neutral or slightly negative when administered intravenously, followed by a switch to a positive charge once the nanoparticles reach the tumor site, is ideal (68). Camptothecin was released after being cleaved by intracellular ROS. The ROS-sensitive thioketal linker was utilized because cancer cells produce a great deal of reactive oxygen species as a result of hypoxia. The dendrimer–camptothecin conjugate exhibited potent antitumor activity in orthotopic pancreatic cancer cell xenografts (92.8% tumor inhibition rate) compared with the control dendrimer without the ROS-sensitive linker (68.3%) and the FDA-approved first-line therapy gemcitabine (62.2%). The significant cellular uptake and accumulation of the conjugate in desmoplastic tumors may be attributed to its smaller size (18.3 nm) and uptake via caveolae-mediated endocytosis and transcytosis [178].

Almawash et al. also investigated the anti-cancer effect of polymeric conjugates of docetaxel and cyclopamine in primary and metastatic pancreatic cancer [195]. Cyclopamine is a steroidal alkaloid inhibitor of the hedgehog (Hh) pathway. The Hh pathway is important for pancreatic tumor progression and metastasis. Docetaxel, on the other hand, is a microtubule stabilizer that binds to β-tubulin, causing mitotic arrest and leading to the death of cancer cells [38,50,195]. The drug conjugates were prepared by covalently linking each drug with methoxy poly (ethylene glycol)-block-poly (2-methyl-2-carboxyl-propylene carbonate via carbodiimide chemistry. The average particle size of each of the conjugates were 73.11 nm (cyclopamine) and 66.28 nm (docetaxel). The combination of the two polymeric conjugates exhibited significant intra-tumoral accumulation and inhibited tumor progression. Additionally, the conjugates were well-tolerated in contrast to the free drugs, which cause severe side effects such as hypersensitivities and peripheral neuropathies.

Despite promising results from various preclinical studies that have investigated the effectiveness of polymer–drug conjugates in treating pancreatic cancer, their clinical translation remains limited.

### 4.2. Antibody-Drug Conjugates for Pancreatic Cancer Treatment

Monoclonal antibodies have been developed successfully for the treatment of a variety of cancers and more than 30 monoclonal antibodies have been approved for the treatment of several cancers in the United States [196]. Antibody–drug conjugates (ADCs) use antibodies that target specific antigens on the surface of tumor cells which are either not present or expressed at low levels in healthy cells to deliver cytotoxic agents. This results in increased selectivity for tumor cells, thereby facilitating active cancer targeting [197,198]. In addition to full-length antibodies, smaller antibody fragments such as fragment antigen binding (Fab), single-chain fragment variable (scFv), and single-domain antibodies are used in drug delivery. The smaller fragments are easier to produce and can potentially penetrate tissues more effectively than full-length antibodies [196,199,200]. To prepare ADCs, antibodies are conjugated to cytotoxic drugs via a suitable linker (Figure 6b) [197,198]. ADCs minimize off-target toxicity and improve cellular uptake of anticancer agents [198,199,201].

Nagaoka and colleagues investigated the anti-tumor activity of a novel ADC, SNS-622-emtansine in pancreatic adenocarcinoma. SNS-622 is an antibody specific to aspartate-β-hydroxylase (ASPH), a type II transmembrane protein overexpressed in pancreatic adenocarcinomas. Overexpression of ASPH promotes the proliferation, migration, invasion, and metastases of pancreatic adenocarcinoma. The ADC exhibited specificity for ASPH and inhibited the growth of the primary tumor and metastatic spread to the lung [201]. In another study, the anti-tumor effect of anti-glypican-1 antibody–drug (monomethyl auristatin F) conjugate was evaluated using pancreatic cancer cell lines [202]. Glypican-1, which promotes rapid cancer proliferation, has been reported to be overabundant in most primary pancreatic adenocarcinoma and it is correlated with poor prognosis. The formulation elicited significant internalization and tumor growth inhibition of glypican-1-positive pancreatic cancer cells [202]. Additionally, Huang et al. developed an innovative ADC, ICAM1-antibody conjugated with mertansine via a succinimidyl 4-(N-maleimidomethyl) cyclohexane-1-carboxylate (SMCC) linker [203]. ICAM1 is a transmembrane glycoprotein overexpressed in pancreatic cancer and it is associated with poor prognosis. The fabricated ADC exhibited considerable preclinical accumulation within the tumor tissues and induced tumor regression [203].

Despite several preclinical investigations that have been conducted on antibody–drug conjugates in pancreatic adenocarcinomas, no compelling anti-cancer effects have been observed clinically and none have been approved for the treatment of pancreatic adenocarcinoma. The FDA has approved about 12 ADC cancer therapies for both solid tumors and hematologic cancers [204]. Only about five antibody–drug conjugates are currently in clinical trials for the treatment of pancreatic cancer (Table 2). The failure of the antibody–drug conjugates in pancreatic adenocarcinoma may be attributed to desmoplasia, which significantly impedes the access of antibody–drug conjugates to pancreatic tumor cells as a consequence of their large molecular size [58]. In addition, pancreatic adenocarcinoma exhibits heterogeneity in the expression of antigens, not only across different patients but also within the same patient over time, which may contribute to treatment failure [200,205]. Therefore, it is recommended that smaller antibody fragments be used, such as the single-chain fragment variable, and single-domain antibodies for the development of ADCs for pancreatic cancer therapy [36,205]. Recent publications by Drago et al. and Marei et al. provided detailed information on antibody–drug conjugates and their impact on cancer therapy [206,207].

### 4.3. Peptide–Drug Conjugates for the Treatment of Pancreatic Cancer

Peptide–drug conjugates have been reviewed elsewhere [188,189,190,208]. Briefly, peptide–drug conjugates are a type of drug delivery system characterized by covalent conjugation of pharmaceutically active agents to a peptide sequence via a suitable linker (Figure 6c). Peptide–drug conjugates are biocompatible and biodegradable and do not exhibit immunogenicity [188,190]. This delivery approach, like other drug conjugates, can be used to alter drugs’ pharmacokinetic characteristics and ensure specific targeting. Peptide–drug conjugates are an emerging delivery approach in the treatment of several cancer types, including pancreatic adenocarcinoma [188,209]. The smaller size of peptide–drug conjugates makes it easier for them to penetrate the refractory tumor microenvironment of pancreatic cancer and micrometastatic tumors compared with nanoparticulate drug delivery and antibody–drug conjugates [45,189]. The average size of an IgG antibody is >1000 amino acids (150 kDa) whereas the length of a peptide used in cancer targeting is between 5 and 25 amino acids (2–5 kDa) [189]. Peptides used in the design of peptide–drug conjugates are broadly categorized into two types: targeting peptides and cell-penetrating peptides [210,211].

Cell-penetrating peptides (CPPs) such as HIV transactivator of transcription (TAT) peptides, octaarginine (R8), and transportan are groups of peptides consisting of fewer than 30 amino acids. Because they are effectively internalized into cells, they have exhibited improved cellular drug uptake in a variety of cancer types. [210]. Cell-penetrating peptides can deliver payloads, such as small molecule drugs, nanoparticles, proteins, and nucleic acids, conjugated to them [212]. However, due to their limited cell selectivity and non-specific cellular uptake, CPPs are not as commonly used as targeting peptides [210,213,214]. Negatively-charged cell-penetrating peptides are more tumor-specific and hence more frequently used than the cationic types [209,212].

Cell-targeting peptides are a group of peptides that can internalize and selectively target cells or tissues [211]. Cell-targeting peptides also function as penetrating peptides and are smaller in size with amino acids ranging from 3 to 14 amino. These targeting peptides such as iRGD (cyclic CRGDKGPDC), iNGR (CRNGRGPDC), somatostatin, and CKAAKN, used in peptide–drug conjugates, are highly specific and selective in targeting some overexpressed ECM components, integrin receptors, EGFR and amino–peptidase N receptor [7,30,181,190]. RGD (arginine-glycine-aspartic acid) is the most common tumor-homing peptide motif widely used in the development of drug conjugates for different cancer types because of the overexpression of integrin receptors in tumor cells [209]. Integrins regulate tumor progression and infiltration into the blood or lymphatic vessels [215,216,217]. Integrin receptors have eight subtypes: ανβ1, ανβ3, ανβ5, ανβ6, ανβ8, α5β1, α8β1and αIIbβ3, out of which ανβ3, ανβ5, α5β1, and ανβ6 are implicated in cancer progression and metastasis [188,216]. However, the use of typical RGD is limited in drug delivery designs because it cannot efficiently penetrate extravascular tumor parenchyma [38].

As an improvement over RGD, a disulfide-based cyclic iRGD has attracted significant attention because it facilitates enhanced penetration and cellular uptake of a variety of therapeutics in various cancer types [8,38,217,218]. The binding of the peptide to β5 integrins stimulates the cleavage and release of the c-terminal sequence, which then interacts with the neuropilin-1 receptor, resulting in the activation of an endocytic transcytosis and trans-tissue transport, which aid the delivery of therapeutic agents [38,94,218]. iRGD-mediated targeting is attractive in pancreatic adenocarcinoma because the iRGD peptide facilitates the penetration of anti-cancer drugs in blood vessels that are involved in tumor growth. It works by increasing the permeability of these blood vessels, making it easier for drugs to reach and effectively target the tumor [217,219]. In addition, it has been demonstrated that iRGD peptide binding to integrin receptors can prevent the expression of ECM glycoproteins such as fibronectin and fibrinogen, resulting in reduced cell adhesion and tumor growth. The potential of polymeric and liposomal iRGD conjugates have been proven in various malignancies such as breast and prostate cancer. It has been shown to exhibit enhanced penetration and accumulation of anticancer agents compared to the naked nanoparticle form [188,220].

Another common tumor-homing peptide used in peptide–drug conjugate is somatostatin. Somatostatin identifies and binds to the somatostatin receptor (SSTRI-5). The interaction of somatostatin and its receptors regulates the uptake and cellular internalization of payloads and also has antisecretory and antiproliferative properties. Somatostatin receptors are found in various neuroendocrine cancers such as neuroendocrine pancreatic, breast, lung, and ovarian cancers [221]. Ragozin and colleagues synthesized somatostatin-derived cyclic peptides conjugated with three different drugs: camptothecin, combretastatin-4A, and azatoxin [221]. All the drug conjugates specifically accumulated within tumors and elicited significant anti-tumor effects in pancreatic cancer cell lines tested [221]. Other tumor-homing peptides are epidermal growth factor protein, gonadotropin-releasing hormones, and angiopep-2 [15,30,212].

The use of peptide-drug conjugates in cancer treatment is still in its infancy. The FDA-approved ^177^lu-dotatate (Lutathera^TM^), used in the treatment of neuroendocrine cancers, is a peptide–drug conjugate. Another peptide–drug conjugate, melfluten, was approved for the treatment of refractory multiple myeloma until it was recently delisted due to its failure in phase III clinical trials [214]. In a recent phase I clinical trial by Dean and colleagues, CEND-1 (iRGD) plus gemcitabine/nab-paclitaxel achieved a response rate of 59% and a median overall survival of 13.2 months in 93% of metastatic pancreatic adenocarcinoma patients [222], compared with a median overall survival of 8.5 months in gemcitabine/nab-paclitaxel group reported by Von Hoff et al. in a phase III clinical trial [23,223]. Additionally, Dókus et al. reported the development of effective Ser-Lys-Ala-Ala-Lys-Asn (SKAAKN) peptide-daunomycin conjugates for selective targeting in PANC-1 pancreatic cancer [30]. The tumor-homing peptide, SKAAKN, was conjugated to daunomycin via a cathepsin B cleavable peptide, GFLG. The peptide–drug conjugates exhibited significant tumor growth inhibition and did not show any toxicity compared with the free drug daunomycin in PANC-1 xenograft model [30]. Peptide–drug conjugates could indeed be a promising strategy for the treatment of pancreatic adenocarcinoma and should be further explored.

## 5. Multistage Delivery Strategy

Multistage drug delivery refers to a stimuli-responsive drug delivery system that involves the sequential release of drugs at different stages of the disease [224]. The objective of this strategy is to enhance the therapeutic results while mitigating the drawbacks of conventional drug delivery systems, which include inadequate drug accumulation at the intended site, rapid clearance from the body, and toxicity to non-targeted areas. This is achieved through the design of a stimuli-responsive system that is expected to disassemble into particles of different sizes, shapes, or surface charges that enable site-specific localization of cytotoxic agents [224,225]. This is different from the functionalized single nanoparticle system in terms of functions and method of preparation. The multistage drug delivery system typically consists of a carrier containing cytotoxic agents embedded in or conjugated to other carrier(s) fabricated to deliver cytotoxic agents to a specific target. [226,227].

**Figure 7:**
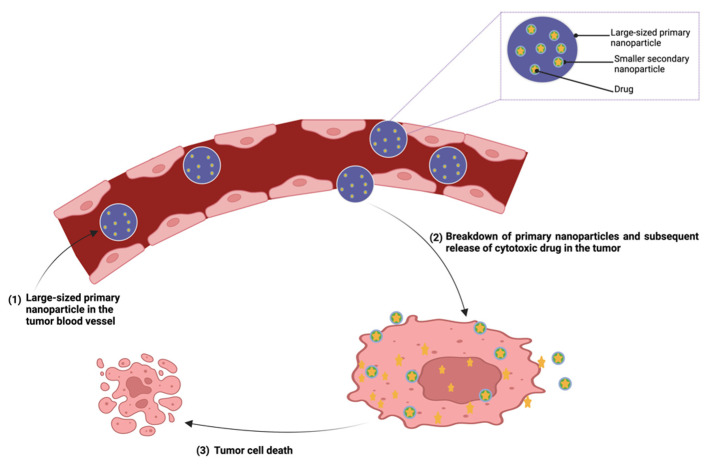
Multistage delivery strategy. The primary nanoparticle delivers the secondary nanoparticles containing the cytotoxic payloads to the tumor microenvironment via the EPR effect. Exposure of the primary nanoparticles to acidic pH or specific enzymes in the tumor microenvironment leads to their breakdown into secondary nanoparticles and subsequent drug release in the tumor. Created with BioRender.com (accessed on 24 February 2023).

The design of the delivery system involves preparing a primary particle that serves as the carrier in which secondary nanoparticles containing anticancer agents are enclosed [228,229]. Following administration and upon exposure of the primary nanoparticles to stimuli such as changes in pH or the presence of specific enzymes in the tumor microenvironment, they break down and release the secondary nanoparticles containing anticancer agent(s) (Figure 7). The initial nano-sized product made of nanoparticles, liposomes, mesoporous silicon particles, and several other nanocarriers can be designed to ensure preferential accumulation in the solid tumors via the EPR effect while the smaller secondary construct can penetrate deeper in the tumor and, if targeted, be internalized through receptor-mediated endocytosis for deep penetration [226,230]. For instance, Wong et al. fabricated multistage quantum dot nanoparticles with an initial size of 100 nm [230]. On reaching the tumor microenvironment after administration, it undergoes size reduction to a smaller size, 10 nm, triggered by matrix metalloproteinases. Their results show that the strategy enhanced penetration throughout the tumor interstitial space [230].

Similarly, Liang et al. demonstrated the effectiveness of multistage drug delivery systems in HER2-overexpressing breast cancer [231]. The study developed a novel lipid envelope-based nanovehicle with a core shell structure containing cascaded aptamers for stepwise drug release and reduced toxicity. The nanovehicle was loaded with naturally occurring anticancer drug epigallocatechin gallate bound to ATP (adenosine-5’-triphosphate) aptamer, forming a ternary complex, and an amino-functionalized lipid matrix as the protective shell. The aptamer HB5 was cross-linked to the nanostructured lipid carrier for specific recognition of HER2 receptors overexpressed in breast cancer. After internalization into tumor cells, protamine facilitates the transfer and escapes from the endosome to cytoplasm and ATP aptamer recognizes and disrupts the ternary complex, leading to EGCG release and ultimately causing tumor death.

Although the multistage delivery strategy dates back over four decades, its potential for pancreatic cancer treatment remains largely unexplored [224]. Given the specific characteristics of this type of cancer, including the dense stroma that poses significant obstacles to drug delivery, utilizing a multistage design could offer greater versatility and significant promise for drug delivery. It has been demonstrated that decreasing the size of nanoparticles may enhance cancer cell targeting; however, this may also lead to rapid elimination following intravenous administration. Hence, it is essential to achieve a balance and ensure that drug delivery systems are neither too small to be rapidly cleared from circulation nor too large to hinder uptake by tumor cells. The use of a multistage design strategy can also be engineered to modify the physicochemical characteristics of nanoparticles, such as their surface charge and shape, all of which impact the overall efficiency of the delivery systems [232]. To ensure the development of optimal drug delivery systems, it is necessary to explore further the potential of multistage design applications in cancer research [224,233]. In a study conducted by [234], multifunctional, size-switchable nanoparticles that improved deep tissue penetration, had optimal intracellular release, and significant antitumor effect in stroma-rich pancreatic and breast cancer models were prepared. Similarly, Li et al. synthesized an ultra-pH-sensitive size-switchable nanoproduct which exhibited improved tumor penetration and therapeutic efficacy [5,235].

## 6. Conclusions

The use of nano-delivery systems has emerged as the long-awaited solution to the limitations of the conventional use of chemotherapeutics. Unfortunately, pancreatic cancers have remained recalcitrant to these “promising products” and no substantial improvements have been observed in overall patient survival. Despite some encouraging preclinical results elicited by these therapies, there has been no significant breakthrough in pancreatic cancer treatment. Desmoplasia in pancreatic cancer impedes penetration and accumulation of anticancer agents and contributes significantly to the poor prognosis of pancreatic cancer. Other factors contributing to poor therapeutic outcomes are the presence of tumor suppressor gene mutations and micro-sized metastatic tumors which are usually impermeable to large molecules, as well as heterogeneity of tumor cells. As a result, regardless of targeting strategies, the efficacy of nanoproducts has been limited by their low penetration and intratumor accumulation.

Notably, the large disparity between preclinical and clinical trial findings may be attributed to vast differences in the physiology and anatomical structure of humans and study animals. Preclinical studies provide valuable insights into the potential efficacy and safety of new drugs or therapies but may be unreliable and difficult to directly extrapolate such results to human patients. Additionally, animal models are usually homogenous compared to the complexity involved in human studies. The main goal in pancreatic cancer drug delivery is to ensure optimal drug delivery by enhancing deep penetration and internalization. These objectives can be achieved by designing delivery approaches that ensure penetration, intracellular uptake, and significant accumulation of cytotoxic agents at the tumor sites.

Based on our understanding of the features of pancreatic cancer and the design of drug delivery systems, the targeting of proteolytic enzymes overexpressed in the pancreatic tumor microenvironment via drug-conjugates such as peptide–drug conjugates and multi-stage drug delivery system approaches may effectively increase drug internalization, accumulation, and overall antitumor activity because of the small particle size of the delivery systems. Although multistage drug delivery approaches have been widely employed in nanotechnology, the approach should be further exploited in combination with small-sized drug conjugates. Furthermore, to limit discrepancies between preclinical and clinical findings and facilitate clinical translation, experimental models such as the genetically engineered mouse models that adequately reflect the unique features of pancreatic adenocarcinoma should be utilized at the preclinical stage.

## Figures and Tables

**Table 1:** Nanoparticle delivery systems in clinical trials for the treatment of pancreatic cancer.

Anticancer Agent	Molecular Target	Phase	Sponsor	ClinicalTrials.gov Identifier
Curcumin and doxorubicin (Imx-110)	Stat3/NF-kB/poly-tyrosine kinase/topoisomerase II	1/2a	Immix Biopharma Australia Pty Ltd.	NCT03382340
Inorganic hafnium oxide (NBTXR3)	Radiation	1	M.D. Anderson Cancer Center	NCT04484909
AGuIX-NP (Theranostic agent)	EPR effect	1/2	Dana-Farber Cancer Institute	NCT04789486

**Table 2:** Antibody–drug conjugates in clinical trials for the treatment of pancreatic cancer.

Anticancer Agent	Molecular Target	Phase	Sponsor	ClinicalTrials.gov Identifier
Monomethyl auristatin E (TORL-2-307-ADC)	Claudin 18.2	1	TORL Biotherapeutics, LLC	NCT05156866
Anthracycline PNU-159682 (SOT102)	Claudin 18.2	1/2	SOTIO Biotech	NCT05525286
Auristatin moiety (A166)	* HER2	1/2	Klus Pharma Inc.	NCT03602079
Monomethyl auristatin E (XB002)	Tissue factor	1	Exelixis	NCT04925284
Duocarmycin analog (vobramitamab duocarmazine)	B7-homolog 3	1	MacroGenics	NCT05293496

* HER2: Human epidermal growth factor receptor 2.

## Data Availability

Not applicable.

## References

[1] Ebelt N.D., Zamloot V., Manuel E.R. (2020). Targeting desmoplasia in pancreatic cancer as an essential first step to effective therapy. Oncotarget.

[2] Yu Q., Qiu Y., Li J., Tang X., Wang X., Cun X., Xu S., Liu Y., Li M., Zhang Z. (2020). Targeting cancer-associated fibroblasts by dual-responsive lipid-albumin nanoparticles to enhance drug perfusion for pancreatic tumor therapy. J. Control. Release.

[3] Lu T., Prakash J. (2021). Nanomedicine Strategies to Enhance Tumor Drug Penetration in Pancreatic Cancer. Int. J. Nanomed..

[4] Siegel R.L., Miller K.D., Fuchs H.E., Jemal A. (2021). Cancer statistics. CA Cancer J. Clin..

[5] Zhao X., Yang X., Wang X., Zhao X., Zhang Y., Liu S., Anderson G.J., Kim S.-J., Li Y., Nie G. (2021). Penetration Cascade of Size Switchable Nanosystem in Desmoplastic Stroma for Improved Pancreatic Cancer Therapy. ACS Nano.

[6] Kędzierska-Kapuza K., Witkowski G., Baumgart-Gryn K., Szylińska A., Durlik M. (2022). Impact of COVID-19 on pancreatic cancer surgery: A high-volume Polish center experience. Adv. Clin. Exp. Med..

[7] Li Y., Zhao Z., Liu H., Fetse J.P., Jain A., Lin C.-Y., Cheng K. (2019). Development of a tumor-responsive nanopolyplex targeting pancreatic cancer cells and stroma. ACS Appl. Mater. Interfaces.

[8] Hosein A.N., Brekken R.A., Maitra A. (2020). Pancreatic cancer stroma: An update on therapeutic targeting strategies. Nat. Rev. Gastroenterol. Hepatol..

[9] Xia C., Dong X., Li H., Cao M., Sun D., He S., Yang F., Yan X., Zhang S., Li N. (2022). Cancer statistics in China and United States, 2022: Profiles, trends, and determinants. Chin. Med. J..

[10] Ferlay J., Partensky C., Bray F. (2016). More deaths from pancreatic cancer than breast cancer in the EU by 2017. Acta Oncol. Stockh. Swed..

[11] Kokkinos J., Ignacio R.M., Sharbeen G., Boyer C., Gonzales-Aloy E., Goldstein D., McCarroll J.A., Phillips P.A. (2020). Australian Pancreatic Cancer Genome Initiative. Targeting the undruggable in pancreatic cancer using nano-based gene silencing drugs. Biomaterials.

[12] Bazeed A.Y., Day C.M., Garg S. (2022). Pancreatic Cancer: Challenges and Opportunities in Locoregional Therapies. Cancers.

[13] Kirkegård J., Bojesen A.B., Nielsen M.F., Mortensen F.V. (2022). Trends in pancreatic cancer incidence, characteristics, and outcomes in Denmark 1980–2019: A nationwide cohort study. Cancer Epidemiol..

[14] Hidalgo M., Cascinu S., Kleeff J., Labianca R., Löhr J.-M., Neoptolemos J., Real F.X., Van Laethem J.-L., Heinemann V. (2015). Addressing the challenges of pancreatic cancer: Future directions for improving outcomes. Pancreatology.

[15] Elechalawar C.K., Hossen N., Shankarappa P., Peer C.J., Figg W.D., Robertson J.D., Bhattacharya R., Mukherjee P. (2020). Targeting Pancreatic Cancer Cells and Stellate Cells Using Designer Nanotherapeutics in vitro. Int. J. Nanomed..

[16] Carvalho T.M.A., Di Molfetta D., Greco M.R., Koltai T., Alfarouk K.O., Reshkin S.J., Cardone R.A. (2021). Tumor Microenvironment Features and Chemoresistance in Pancreatic Ductal Adenocarcinoma: Insights into Targeting Physicochemical Barriers and Metabolism as Therapeutic Approaches. Cancers.

[17] Pontious C., Kaul S., Hong M., Hart P.A., Krishna S.G., Lara L.F., Conwell D.L., Cruz-Monserrate Z. (2019). Cathepsin E expression and activity: Role in the detection and treatment of pancreatic cancer. Pancreatology.

[18] Cannon A., Thompson C., Hall B.R., Jain M., Kumar S., Batra S.K. (2018). Desmoplasia in pancreatic ductal adenocarcinoma: Insight into pathological function and therapeutic potential. Genes Cancer.

[19] Conte M., Cauda V. (2022). Multimodal Therapies against Pancreatic Ductal Adenocarcinoma: A Review on Synergistic Approaches toward Ultimate Nanomedicine Treatments. Adv. Ther..

[20] Badger S., Brant J., Jones C., McClements J., Loughrey M., Taylor M., Diamond T., McKie L. (2010). The role of surgery for pancreatic cancer: A 12-year review of patient outcome. Ulst. Med. J..

[21] Tomasello G., Ghidini M., Costanzo A., Ghidini A., Russo A., Barni S., Passalacqua R., Petrelli F. (2019). Outcome of head compared to body and tail pancreatic cancer: A systematic review and meta-analysis of 93 studies. J. Gastrointest. Oncol..

[22] Kleeff J., Korc M., Apte M., La Vecchia C., Johnson C.D., Biankin A.V., Neale R.E., Tempero M., Tuveson D.A., Hruban R.H. (2016). Pancreatic cancer. Nat. Rev. Dis. Prim..

[23] Diab M., Azmi A., Mohammad R., Philip P.A. (2019). Pharmacotherapeutic strategies for treating pancreatic cancer: Advances and challenges. Expert Opin. Pharmacother..

[24] Lee M., Kwon W., Kim H., Byun Y., Han Y., Kang J.S., Choi Y.J., Jang J.-Y. (2020). The Role of Location of Tumor in the Prognosis of the Pancreatic Cancer. Cancers.

[25] Wang S., Zheng Y., Yang F., Zhu L., Zhu X.-Q., Wang Z.-F., Wu X.-L., Zhou C.-H., Yan J.-Y., Hu B.-Y. (2021). The Molecular Biology of Pancreatic Adenocarcinoma: Translational Challenges and Clinical Perspectives. Signal Transduct. Target. Ther..

[26] Silverman D.T., Hoover R.N., Brown L.M., Swanson G.M., Schiffman M., Greenberg R.S., Hayes R.B., Lillemoe K.D., Schoenberg J.B., Schwartz A.G. (2003). Why do Black Americans have a higher risk of pancreatic cancer than White Americans?. Epidemiology.

[27] Herremans K.M., Riner A.N., Winn R.A., Trevino J.G. (2021). Diversity and Inclusion in Pancreatic Cancer Clinical Trials. Gastroenterology.

[28] Moniri M.R., Dai L.-J., Warnock G.L. (2014). The challenge of pancreatic cancer therapy and novel treatment strategy using engineered mesenchymal stem cells. Cancer Gene Ther..

[29] Whatcott C.J., Diep C.H., Jiang P., Watanabe A., LoBello J., Sima C., Hostetter G., Shepard H.M., Von Hoff D.D., Han H. (2015). Desmoplasia in Primary Tumors and Metastatic Lesions of Pancreatic Cancerfibrosis in Pancreatic Metastases. Clin. Cancer Res..

[30] Dókus L.E., Lajkó E., Ranđelović I., Mező D., Schlosser G., Kőhidai L., Tóvári J., Mező G. (2020). Phage Display-Based Homing Peptide-Daunomycin Conjugates for Selective Drug Targeting to PANC-1 Pancreatic Cancer. Pharmaceutics.

[31] Tarannum M., Vivero-Escoto J.L. (2022). Nanoparticle-based therapeutic strategies targeting major clinical challenges in pancreatic cancer treatment. Adv. Drug Deliv. Rev..

[32] Zhu H., Li T., Du Y., Li M. (2018). Pancreatic cancer: Challenges and opportunities. BMC Med..

[33] Manrai M., Tilak T.V.S.V.G.K., Dawra S., Srivastava S., Singh A. (2021). Current and emerging therapeutic strategies in pancreatic cancer: Challenges and opportunities. World J. Gastroenterol..

[34] Zhang L., Sanagapalli S., Stoita A. (2018). Challenges in diagnosis of pancreatic cancer. World J. Gastroenterol..

[35] Li J., Peng L., Chen Q., Ye Z., Zhao T., Hou S., Gu J., Hang Q. (2022). Integrin β1 in Pancreatic Cancer: Expressions, Functions, and Clinical Implications. Cancers.

[36] Liu L., Kshirsagar P.G., Gautam S.K., Gulati M., Wafa E.I., Christiansen J.C., White B.M., Mallapragada S.K., Wannemuehler M.J., Kumar S. (2022). Nanocarriers for pancreatic cancer imaging, treatments, and immunotherapies. Theranostics.

[37] Khan I.U., Serra C.A., Anton N., Vandamme T. (2013). Microfluidics: A focus on improved cancer targeted drug delivery systems. J. Control. Release.

[38] Merika E.E., Syrigos K.N., Saif M.W. (2012). Desmoplasia in pancreatic cancer. Can we fight it?. Gastroenterol. Res. Pract..

[39] Adesina S.K., Holly A., Kramer-Marek G., Capala J., Akala E.O. (2014). Polylactide-Based Paclitaxel-Loaded Nanoparticles Fabricated by Dispersion Polymerization: Characterization, Evaluation in Cancer Cell Lines, and Preliminary Biodistribution Studies. J. Pharm. Sci..

[40] Khare V., Alam N., Saneja A., Dubey R.D., Gupta P.N. (2014). Targeted Drug Delivery Systems for Pancreatic Cancer. J. Biomed. Nanotechnol..

[41] Longnecker D.S. (2014). Anatomy and Histology of the Pancreas (version 1.0). Pancreapedia Exocrine Pancreas Knowl. Base.

[42] Cesmebasi A., Malefant J., Patel S.D., Du Plessis M., Renna S., Tubbs R.S., Loukas M. (2015). The surgical anatomy of the lymphatic system of the pancreas. Clin. Anat..

[43] van Erning F.N., Mackay T.M., van der Geest L.G., Groot Koerkamp B., van Laarhoven H.W., Bonsing B.A., Wilmink J.W., van Santvoort H.C., de Vos-Geelen J., van Eijck C.H.J. (2018). Association of the location of pancreatic ductal adenocarcinoma (head, body, tail) with tumor stage, treatment, and survival: A population-based analysis. Acta Oncol..

[44] Artinyan A., Soriano P.A., Prendergast C., Low T., Ellenhorn J.D., Kim J. (2008). The anatomic location of pancreatic cancer is a prognostic factor for survival. HPB.

[45] Jiang S., Fagman J.B., Ma Y., Liu J., Vihav C., Engstrom C., Liu B., Chen C. (2022). A comprehensive review of pancreatic cancer and its therapeutic challenges. Aging.

[46] Masugi Y. (2022). The Desmoplastic Stroma of Pancreatic Cancer: Multilayered Levels of Heterogeneity, Clinical Significance, and Therapeutic Opportunities. Cancers.

[47] Falasca M., Kim M., Casari I. (2016). Pancreatic cancer: Current research and future directions. Biochim. et Biophys. Acta (BBA)-Rev. Cancer.

[48] Han H., Hou Y., Chen X., Zhang P., Kang M., Jin Q., Ji J., Gao M. (2020). Metformin-Induced Stromal Depletion to Enhance the Penetration of Gemcitabine-Loaded Magnetic Nanoparticles for Pancreatic Cancer Targeted Therapy. J. Am. Chem. Soc..

[49] Dimastromatteo J., Houghton J.L., Lewis J.S., Kelly K.A. (2015). Challenges of Pancreatic Cancer. Cancer J..

[50] Schnittert J., Bansal R., Prakash J. (2019). Targeting Pancreatic Stellate Cells in Cancer. Trends Cancer.

[51] Miao L., Liu Q., Lin C.M., Luo C., Wang Y., Liu L., Yin W., Hu S., Kim W.Y., Huang L. (2017). Targeting Tumor-Associated Fibroblasts for Therapeutic Delivery in Desmoplastic TumorsIn Situ Generation of Tumor-Suppressive Fibroblasts. Cancer Res..

[52] Whittle M.C., Hingorani S.R. (2019). Fibroblasts in Pancreatic Ductal Adenocarcinoma: Biological Mechanisms and Therapeutic Targets. Gastroenterology.

[53] Norton J., Foster D., Chinta M., Titan A., Longaker M. (2020). Pancreatic Cancer Associated Fibroblasts (CAF): Under-Explored Target for Pancreatic Cancer Treatment. Cancers.

[54] Nandi T., Pradyuth S., Singh A.K., Chitkara D., Mittal A. (2020). Therapeutic agents for targeting desmoplasia: Current status and emerging trends. Drug Discov. Today.

[55] Polani F., Grierson P.M., Lim K.-H. (2021). Stroma-targeting strategies in pancreatic cancer: Past lessons, challenges and prospects. World J. Gastroenterol..

[56] Sivapalan L., Kocher H., Ross-Adams H., Chelala C. (2021). Molecular profiling of ctDNA in pancreatic cancer: Opportunities and challenges for clinical application. Pancreatology.

[57] Chitkara D., Mittal A., Behrman S.W., Kumar N., Mahato R.I. (2013). Self-assembling, amphiphilic polymer–gemcitabine conjugate shows enhanced antitumor efficacy against human pancreatic adenocarcinoma. Bioconjugate Chem..

[58] Boyd L.N., Andini K.D., Peters G.J., Kazemier G., Giovannetti E. (2022). Heterogeneity and plasticity of cancer-associated fibroblasts in the pancreatic tumor microenvironment. Seminars in Cancer Biology.

[59] Ren B., Cui M., Yang G., Wang H., Feng M., You L., Zhao Y. (2018). Tumor microenvironment participates in metastasis of pancreatic cancer. Mol. Cancer.

[60] Ho W.J., Jaffee E.M., Zheng L. (2020). The tumour microenvironment in pancreatic cancer—Clinical challenges and opportunities. Nat. Rev. Clin. Oncol..

[61] Stine Z., Altman B., Hsieh A., Gouw A., Dang C. (2014). Deregulation of the Cellular Energetics of Cancer Cells. Pathobiology of Human Disease.

[62] Chen X., Zhou W., Liang C., Shi S., Yu X., Chen Q., Sun T., Lu Y., Zhang Y., Guo Q. (2019). Codelivery Nanosystem Targeting the Deep Microenvironment of Pancreatic Cancer. Nano Lett..

[63] Bannoura S.F., Uddin H., Nagasaka M., Fazili F., Al-Hallak M.N., Philip P.A., El-Rayes B., Azmi A.S. (2021). Targeting KRAS in pancreatic cancer: New drugs on the horizon. Cancer Metastasis Rev..

[64] Nakajima E.C., Drezner N., Li X., Mishra-Kalyani P.S., Liu Y., Zhao H., Bi Y., Liu J., Rahman A., Wearne E. (2022). FDA Approval Summary: Sotorasib for *KRAS G12C*-Mutated Metastatic NSCLC. Clin. Cancer Res..

[65] Strickler J.H., Satake H., George T.J., Yaeger R., Hollebecque A., Garrido-Laguna I., Schuler M., Burns T.F., Coveler A.L., Falchook G.S. (2023). Sotorasib in KRAS p. G12C–Mutated Advanced Pancreatic Cancer. N. Engl. J. Med..

[66] Khawar I.A., Kim J.H., Kuh H.-J. (2015). Improving drug delivery to solid tumors: Priming the tumor microenvironment. J. Control. Release.

[67] Maeda H., Tsukigawa K., Fang J. (2016). A Retrospective 30 Years After Discovery of the Enhanced Permeability and Retention Effect of Solid Tumors: Next-Generation Chemotherapeutics and Photodynamic Therapy-Problems, Solutions, and Prospects. Microcirculation.

[68] Ejigah V., Owoseni O., Bataille-Backer P., Ogundipe O.D., Fisusi F.A., Adesina S.K. (2022). Approaches to Improve Macromolecule and Nanoparticle Accumulation in the Tumor Microenvironment by the Enhanced Permeability and Retention Effect. Polymers.

[69] Greish K. (2012). Enhanced permeability and retention effect for selective targeting of anticancer nanomedicine: Are we there yet?. Drug Discov. Today Technol..

[70] Fang J., Nakamura H., Maeda H. (2011). The EPR effect: Unique features of tumor blood vessels for drug delivery, factors involved, and limitations and augmentation of the effect. Adv. Drug Deliv. Rev..

[71] Natfji A.A., Ravishankar D., Osborn H.M.I., Greco F. (2017). Parameters Affecting the Enhanced Permeability and Retention Effect: The Need for Patient Selection. J. Pharm. Sci..

[72] Maeda H., Bharate G., Daruwalla J. (2009). Polymeric drugs for efficient tumor-targeted drug delivery based on EPR-effect. Eur. J. Pharm. Biopharm..

[73] Kalyane D., Raval N., Maheshwari R., Tambe V., Kalia K., Tekade R.K. (2019). Employment of enhanced permeability and retention effect (EPR): Nanoparticle-based precision tools for targeting of therapeutic and diagnostic agent in cancer. Mater. Sci. Eng. C.

[74] Rajora A.K., Ravishankar D., Osborn H.M.I., Greco F. (2014). Impact of the Enhanced Permeability and Retention (EPR) Effect and Cathepsins Levels on the Activity of Polymer-Drug Conjugates. Polymers.

[75] Fang J., Islam W., Maeda H. (2020). Exploiting the dynamics of the EPR effect and strategies to improve the therapeutic effects of nanomedicines by using EPR effect enhancers. Adv. Drug Deliv. Rev..

[76] Edwards P., Kang B.W., Chau I. (2021). Targeting the Stroma in the Management of Pancreatic Cancer. Front. Oncol..

[77] Liu X., Jiang J., Meng H. (2019). Transcytosis—An effective targeting strategy that is complementary to “EPR effect” for pancreatic cancer nano drug delivery. Theranostics.

[78] Islam W., Niidome T., Sawa T. (2022). Enhanced Permeability and Retention Effect as a Ubiquitous and Epoch-Making Phenomenon for the Selective Drug Targeting of Solid Tumors. J. Pers. Med..

[79] Nel A., Ruoslahti E., Meng H. (2017). New Insights into “Permeability” as in the Enhanced Permeability and Retention Effect of Cancer Nanotherapeutics. ACS Nano.

[80] Xie Y., Hang Y., Wang Y., Sleightholm R., Prajapati D.R., Bader J., Yu A., Tang W., Jaramillo L., Li J. (2020). Stromal Modulation and Treatment of Metastatic Pancreatic Cancer with Local Intraperitoneal Triple miRNA/siRNA Nanotherapy. ACS Nano.

[81] Pandit S., Dutta D., Nie S. (2020). Active transcytosis and new opportunities for cancer nanomedicine. Nat. Mater..

[82] Zhou Q., Li J., Xiang J., Shao S., Zhou Z., Tang J., Shen Y. (2022). Transcytosis-enabled active extravasation of tumor nanomedicine. Adv. Drug Deliv. Rev..

[83] Tanaka H.Y., Kano M.R. (2018). Stromal barriers to nanomedicine penetration in the pancreatic tumor microenvironment. Cancer Sci..

[84] Wallrapp C., Hähnel S., Müller-Pillasch F., Burghardt B., Iwamura T., Ruthenbürger M., Lerch M.M., Adler G., Gress T.M. (2000). A Novel Transmembrane Serine Protease (TMPRSS3) Overexpressed in Pancreatic Cancer1, 2. Cancer Res..

[85] Herszényi L., Barabás L., Hritz I., István G., Tulassay Z. (2014). Impact of proteolytic enzymes in colorectal cancer development and progression. World J. Gastroenterol..

[86] Uchima Y., Sawada T., Nishihara T., Maeda K., Ohira M., Hirakawa K. (2004). Inhibition and Mechanism of Action of a Protease Inhibitor in Human Pancreatic Cancer Cells. Pancreas.

[87] Mótyán J.A., Tóth F., Tőzsér J. (2013). Research applications of proteolytic enzymes in molecular biology. Biomolecules.

[88] Scott C.J., Taggart C.C. (2010). Biologic protease inhibitors as novel therapeutic agents. Biochimie.

[89] Rudzińska M., Daglioglu C., Savvateeva L.V., Kaci F.N., Antoine R., Zamyatnin A.A. (2021). Current status and perspectives of protease inhibitors and their combination with nanosized drug delivery systems for targeted cancer therapy. Drug Des. Dev. Ther..

[90] Vandooren J., Opdenakker G., Loadman P.M., Edwards D.R. (2016). Proteases in cancer drug delivery. Adv. Drug Deliv. Rev..

[91] Patsouras D., Papaxoinis K., Kostakis A., Safioleas M.C., Lazaris A.C., Nicolopoulou-Stamati P. (2015). Fibroblast activation protein and its prognostic significance in correlation with vascular endothelial growth factor in pancreatic adenocarcinoma. Mol. Med. Rep..

[92] Cohen S.J., Alpaugh R.K., Palazzo I., Meropol N.J., Rogatko A., Xu Z., Hoffman J.P., Weiner L.M., Cheng J.D. (2008). Fibroblast Activation Protein and Its Relationship to Clinical Outcome in Pancreatic Adenocarcinoma. Pancreas.

[93] Keane F.M., Yao T.-W., Seelk S., Gall M.G., Chowdhury S., Poplawski S.E., Lai J.H., Li Y., Wu W., Farrell P. (2014). Quantitation of fibroblast activation protein (FAP)-specific protease activity in mouse, baboon and human fluids and organs. FEBS Open Bio.

[94] Akinboye E.S., Brennen W.N., Rosen D.M., Bakare O., Denmeade S.R. (2016). Iterative design of emetine-based prodrug targeting fibroblast activation protein (FAP) and dipeptidyl peptidase IV DPPIV using a tandem enzymatic activation strategy. Prostate.

[95] Lo A., Li C.-P., Buza E.L., Blomberg R., Govindaraju P., Avery D., Monslow J., Hsiao M., Puré E. (2017). Fibroblast activation protein augments progression and metastasis of pancreatic ductal adenocarcinoma. J. Clin. Investig..

[96] Zhao L., Chen J., Pang Y., Fu K., Shang Q., Wu H., Sun L., Lin Q., Chen H. (2022). Fibroblast activation protein-based theranostics in cancer research: A state-of-the-art review. Theranostics.

[97] Huber M.A., Schubert R.D., Peter R.U., Kraut N., Park J.E., Rettig W.J., Garin-Chesa P. (2003). Fibroblast Activation Protein: Differential Expression and Serine Protease Activity in Reactive Stromal Fibroblasts of Melanocytic Skin Tumors. J. Investig. Dermatol..

[98] Liu R., Li H., Liu L., Yu J., Ren X. (2012). Fibroblast activation protein: A potential therapeutic target in cancer. Cancer Biol. Ther..

[99] Park H., Lee Y., Lee H., Kim J.-W., Hwang J.-H., Kim J., Yoon Y.-S., Han H.-S., Kim H. (2017). The prognostic significance of cancer-associated fibroblasts in pancreatic ductal adenocarcinoma. Tumor Biol..

[100] Lin H.-J., Liang T.-L., Chang Y.-Y., Liu D.-Z., Fan J.-Y., Roffler S.R., Lin S.-Y. (2022). Development of Irinotecan Liposome Armed with Dual-Target Anti-Epidermal Growth Factor Receptor and Anti-Fibroblast Activation Protein-Specific Antibody for Pancreatic Cancer Treatment. Pharmaceutics.

[101] Abd-Elgaliel W.R., Cruz-Monserrate Z., Wang H., Logsdon C.D., Tung C.-H. (2013). Pancreatic cancer-associated Cathepsin E as a drug activator. J. Control. Release.

[102] Jones L., Ghaneh P., Humphreys M., Neoptolemos J.P. (1999). The Matrix Metalloproteinases and Their Inhibitors in the Treatment of Pancreatic Cancer. Ann. N. Y. Acad. Sci..

[103] Ghaneh P., Kawesha A., Evans J.D., Neoptolemos J. (2002). Molecular prognostic markers in pancreatic cancer. J. Hepato-Biliary-Pancreatic Surg..

[104] Han F., Zhu H.-G. (2010). Caveolin-1 Regulating the Invasion and Expression of Matrix Metalloproteinase (MMPs) in Pancreatic Carcinoma Cells. J. Surg. Res..

[105] Kulkarni P.S., Haldar M.K., Nahire R.R., Katti P., Ambre A.H., Muhonen W.W., Shabb J.B., Padi S.K.R., Singh R.K., Borowicz P.P. (2014). MMP-9 Responsive PEG Cleavable Nanovesicles for Efficient Delivery of Chemotherapeutics to Pancreatic Cancer. Mol. Pharm..

[106] Niedergethmann M., Wostbrock B., Sturm J.W., Willeke F., Post S., Hildenbrand R. (2004). Prognostic Impact of Cysteine Proteases Cathepsin B and Cathepsin L in Pancreatic Adenocarcinoma. Pancreas.

[107] Han H., Valdepérez D., Jin Q., Yang B., Li Z., Wu Y., Pelaz B., Parak W.J., Ji J. (2017). Dual Enzymatic Reaction-Assisted Gemcitabine Delivery Systems for Programmed Pancreatic Cancer Therapy. ACS Nano.

[108] Sulpizio S., Franceschini N., Piattelli A., Di Sebastiano P., Innocenti P., Selvaggi F. (2012). Cathepsins and pancreatic cancer: The 2012 update. Pancreatology.

[109] Chu E., Sartorelli A.C. (2018). Cancer chemotherapy. Lange’s Basic and Clinical Pharmacology.

[110] Liu X., Jiang J., Ji Y., Lu J., Chan R., Meng H. (2017). Targeted drug delivery using iRGD peptide for solid cancer treatment. Mol. Syst. Des. Eng..

[111] Arias J.L. (2011). Drug Targeting Strategies in Cancer Treatment: An Overview. Mini-Rev. Med. Chem..

[112] Young K., Hughes D.J., Cunningham D., Starling N. (2018). Immunotherapy and pancreatic cancer: Unique challenges and potential opportunities. Ther. Adv. Med. Oncol..

[113] Fan J.-Q., Wang M.-F., Chen H.-L., Shang D., Das J.K., Song J. (2020). Current advances and outlooks in immunotherapy for pancreatic ductal adenocarcinoma. Mol. Cancer.

[114] Balachandran V.P., Beatty G.L., Dougan S.K. (2019). Broadening the Impact of Immunotherapy to Pancreatic Cancer: Challenges and Opportunities. Gastroenterology.

[115] Bear A.S., Vonderheide R.H., O’Hara M.H. (2020). Challenges and Opportunities for Pancreatic Cancer Immunotherapy. Cancer Cell.

[116] Wu J., Cai J. (2020). Dilemma and Challenge of Immunotherapy for Pancreatic Cancer. Dig. Dis. Sci..

[117] Martinez-Bosch N., Vinaixa J., Navarro P. (2018). Immune Evasion in Pancreatic Cancer: From Mechanisms to Therapy. Cancers.

[118] Torphy R.J., Zhu Y., Schulick R.D. (2018). Immunotherapy for pancreatic cancer: Barriers and breakthroughs. Ann. Gastroenterol. Surg..

[119] Kamath S.D., Kalyan A., Kircher S., Nimeiri H., Fought A.J., Benson A., Mulcahy M. (2020). Ipilimumab and gemcitabine for advanced pancreatic cancer: A phase Ib study. Oncologist.

[120] Wu A.A., Bever K.M., Ho W.J., Fertig E.J., Niu N., Zheng L., Parkinson R.M., Durham J.N., Onners B.L., Ferguson A.K. (2020). A Phase II Study of Allogeneic GM-CSF–Transfected Pancreatic Tumor Vaccine (GVAX) with Ipilimumab as Maintenance Treatment for Metastatic Pancreatic Cancer. Clin. Cancer Res..

[121] Feng M., Xiong G., Cao Z., Yang G., Zheng S., Song X., You L., Zheng L., Zhang T., Zhao Y. (2017). PD-1/PD-L1 and immunotherapy for pancreatic cancer. Cancer Lett..

[122] Ribas A., Puzanov I., Dummer R., Schadendorf D., Hamid O., Robert C., Hodi F.S., Schachter J., Pavlick A.C., Lewis K.D. (2015). Pembrolizumab versus investigator-choice chemotherapy for ipilimumab-refractory melanoma (KEYNOTE-002): A randomised, controlled, phase 2 trial. Lancet Oncol..

[123] Weiss G.J., Blaydorn L., Beck J., Bornemann-Kolatzki K., Urnovitz H., Schütz E., Khemka V. (2018). Phase Ib/II study of gemcitabine, nab-paclitaxel, and pembrolizumab in metastatic pancreatic adenocarcinoma. Investig. New Drugs.

[124] Wainberg Z.A., Hochster H.S., Kim E.J., George B., Kaylan A., Chiorean E.G., Waterhouse D.M., Guiterrez M., Parikh A., Jain R. (2020). Open-label, Phase I Study of Nivolumab Combined with nab-Paclitaxel Plus Gemcitabine in Advanced Pancreatic CancerNivo Plus nab-Pac and Gem in Advanced Pancreatic Cancer. Clin. Cancer Res..

[125] Le D.T., Picozzi V.J., Ko A.H., Wainberg Z.A., Kindler H., Wang-Gillam A., Oberstein P.E., Morse M.A., Zeh H.J., Weekes C.D. (2019). Results from a Phase IIb, Randomized, Multicenter Study of GVAX Pancreas and CRS-207 Compared with Chemotherapy in Adults with Previously Treated Metastatic Pancreatic Adenocarcinoma (ECLIPSE Study). Clin. Cancer Res..

[126] Middleton G., Silcocks P., Cox T., Valle J., Wadsley J., Propper D., Coxon F., Ross P., Madhusudan S., Roques T. (2014). Gemcitabine and capecitabine with or without telomerase peptide vaccine GV1001 in patients with locally advanced or metastatic pancreatic cancer (TeloVac): An open-label, randomised, phase 3 trial. Lancet Oncol..

[127] Di Federico A., Mosca M., Pagani R., Carloni R., Frega G., De Giglio A., Rizzo A., Ricci D., Tavolari S., Di Marco M. (2022). Immunotherapy in Pancreatic Cancer: Why Do We Keep Failing? A Focus on Tumor Immune Microenvironment, Predictive Biomarkers and Treatment Outcomes. Cancers.

[128] Schizas D., Charalampakis N., Kole C., Economopoulou P., Koustas E., Gkotsis E., Ziogas D., Psyrri A., Karamouzis M.V. (2020). Immunotherapy for pancreatic cancer: A 2020 update. Cancer Treat. Rev..

[129] Fan B.-G., Andrén-Sandberg Å. (2007). Photodynamic Therapy for Pancreatic Cancer. Pancreas.

[130] Xie Q., Jia L., Liu Y.-H., Wei C.-G. (2009). Synergetic anticancer effect of combined gemcitabine and photodynamic therapy on pancreatic cancer in vivo. World J. Gastroenterol..

[131] Huggett M.T., Jermyn M., Gillams A., Illing R., Mosse S., Novelli M., Kent E., Bown S.G., Hasan T., Pogue B.W. (2014). Phase I/II study of verteporfin photodynamic therapy in locally advanced pancreatic cancer. Br. J. Cancer.

[132] Lu J., Roy B., Anderson M., Leggett C.L., Levy M.J., Pogue B., Hasan T., Wang K.K. (2019). Verteporfin- and sodium porfimer-mediated photodynamic therapy enhances pancreatic cancer cell death without activating stromal cells in the microenvironment. J. Biomed. Opt..

[133] Wang Y., Wang H., Zhou L., Lu J., Jiang B., Liu C., Guo J. (2020). Photodynamic therapy of pancreatic cancer: Where have we come from and where are we going?. Photodiagn. Photodyn. Ther..

[134] Kim M.M., Darafsheh A. (2020). Light Sources and Dosimetry Techniques for Photodynamic Therapy. Photochem. Photobiol..

[135] Meng Z., Hou W., Zhou H., Zhou L., Chen H., Wu C. (2018). Therapeutic Considerations and Conjugated Polymer-Based Photosensitizers for Photodynamic Therapy. Macromol. Rapid Commun..

[136] Celli J.P., Solban N., Liang A., Pereira S.P., Hasan T. (2011). Verteporfin-based photodynamic therapy overcomes gemcitabine insensitivity in a panel of pancreatic cancer cell lines. Lasers Surg. Med..

[137] Yu X., Zhu W., Di Y., Gu J., Guo Z., Li H., Fu D., Jin C. (2017). Triple-functional albumin-based nanoparticles for combined chemotherapy and photodynamic therapy of pancreatic cancer with lymphatic metastases. Int. J. Nanomed..

[138] Yano T., Wang K.K. (2020). Photodynamic Therapy for Gastrointestinal Cancer. Photochem. Photobiol..

[139] Hafiz S.S., Xavierselvan M., Gokalp S., Labadini D., Barros S., Duong J., Foster M., Mallidi S. (2022). Eutectic Gallium–Indium Nanoparticles for Photodynamic Therapy of Pancreatic Cancer. ACS Appl. Nano Mater..

[140] Farokhzad O.C., Langer R. (2009). Impact of Nanotechnology on Drug Delivery. ACS Nano.

[141] Attia M.F., Anton N., Wallyn J., Omran Z., Vandamme T.F. (2019). An overview of active and passive targeting strategies to improve the nanocarriers efficiency to tumour sites. J. Pharm. Pharmacol..

[142] Sun R., Xiang J., Zhou Q., Piao Y., Tang J., Shao S., Zhou Z., Bae Y.H., Shen Y. (2022). The tumor EPR effect for cancer drug delivery: Current status, limitations, and alternatives. Adv. Drug Deliv. Rev..

[143] Adesina S.K., Akala E.O. (2015). Nanotechnology Approaches for the Delivery of Exogenous siRNA for HIV Therapy. Mol. Pharm..

[144] Alshawwa S.Z., Kassem A.A., Farid R.M., Mostafa S.K., Labib G.S. (2022). Nanocarrier Drug Delivery Systems: Characterization, Limitations, Future Perspectives and Implementation of Artificial Intelligence. Pharmaceutics.

[145] Alshememry A.K., Alsaleh N.B., Alkhudair N., Alzhrani R., Alshamsan A. (2022). Recent nanotechnology advancements to treat multidrug-resistance pancreatic cancer: Pre-clinical and clinical overview. Front. Pharmacol..

[146] Delplace V., Couvreur P., Nicolas J. (2014). Recent trends in the design of anticancer polymer prodrug nanocarriers. Polym. Chem..

[147] Bhattacharjee S. (2021). Understanding the burst release phenomenon: Toward designing effective nanoparticulate drug-delivery systems. Ther. Deliv..

[148] Li S.-D., Huang L. (2010). Stealth nanoparticles: High density but sheddable PEG is a key for tumor targeting. J. Control. Release Off. J. Control. Release Soc..

[149] Amoozgar Z., Yeo Y. (2012). Recent advances in stealth coating of nanoparticle drug delivery systems. WIREs Nanomed. Nanobiotechnol..

[150] Vllasaliu D., Fowler R., Stolnik S. (2014). PEGylated nanomedicines: Recent progress and remaining concerns. Expert Opin. Drug Deliv..

[151] Hoogenboezem E.N., Duvall C.L. (2018). Harnessing albumin as a carrier for cancer therapies. Adv. Drug Deliv. Rev..

[152] Hassanin I., Elzoghby A. (2020). Albumin-based nanoparticles: A promising strategy to overcome cancer drug resistance. Cancer Drug Resist..

[153] Kim B., Lee C., Lee E.S., Shin B.S., Youn Y.S. (2016). Paclitaxel and curcumin co-bound albumin nanoparticles having antitumor potential to pancreatic cancer. Asian J. Pharm. Sci..

[154] An F.-F., Zhang X.-H. (2017). Strategies for Preparing Albumin-based Nanoparticles for Multifunctional Bioimaging and Drug Delivery. Theranostics.

[155] Cheng Z., Huang Y., Shen Q., Zhao Y., Wang L., Yu J., Lu W. (2021). A camptothecin-based, albumin-binding prodrug enhances efficacy and safety in vivo. Eur. J. Med. Chem..

[156] Hirakawa N., Ishima Y., Kinoshita R., Nakano R., Chuang V.T.G., Ando H., Shimizu T., Okuhira K., Maruyama T., Otagiri M. (2021). Reduction-Responsive and Multidrug Deliverable Albumin Nanoparticles: An Antitumor Drug to Abraxane against Human Pancreatic Tumor-Bearing Mice. ACS Appl. Bio Mater..

[157] Tan Y.L., Ho H.K. (2018). Navigating albumin-based nanoparticles through various drug delivery routes. Drug Discov. Today.

[158] Cho H., Jeon S.I., Ahn C.-H., Shim M.K., Kim K. (2022). Emerging Albumin-Binding Anticancer Drugs for Tumor-Targeted Drug Delivery: Current Understandings and Clinical Translation. Pharmaceutics.

[159] Elzoghby A.O., Samy W.M., Elgindy N.A. (2012). Albumin-based nanoparticles as potential controlled release drug delivery systems. J. Control. Release.

[160] Yu X., Jin C. (2016). Application of albumin-based nanoparticles in the management of cancer. J. Mater. Sci. Mater. Med..

[161] Goldstein D., El-Maraghi R.H., Hammel P., Heinemann V., Kunzmann V., Sastre J., Scheithauer W., Siena S., Tabernero J., Teixeira L. (2015). nab-Paclitaxel Plus Gemcitabine for Metastatic Pancreatic Cancer: Long-Term Survival From a Phase III Trial. Gynecol. Oncol..

[162] Feng J., Zhao C., Wang L., Qu L., Zhu H., Yang Z., An G., Tian H., Shou C. (2018). Development of a novel albumin-based and maleimidopropionic acid-conjugated peptide with prolonged half-life and increased in vivo anti-tumor efficacy. Theranostics.

[163] Han H., Wang J., Chen T., Yin L., Jin Q., Ji J. (2017). Enzyme-sensitive gemcitabine conjugated albumin nanoparticles as a versatile theranostic nanoplatform for pancreatic cancer treatment. J. Colloid Interface Sci..

[164] Yue C., Liu P., Zheng M., Zhao P., Wang Y., Ma Y., Cai L. (2013). IR-780 dye loaded tumor targeting theranostic nanoparticles for NIR imaging and photothermal therapy. Biomaterials.

[165] Arias J.L. (2013). Liposomes in drug delivery: A patent review (2007–present). Expert Opin. Ther. Pat..

[166] Bozzuto G., Molinari A. (2015). Liposomes as nanomedical devices. Int. J. Nanomed..

[167] Ji T., Li S., Zhang Y., Lang J., Ding Y., Zhao X., Zhao R., Li Y., Shi J., Hao J. (2016). An MMP-2 Responsive Liposome Integrating Antifibrosis and Chemotherapeutic Drugs for Enhanced Drug Perfusion and Efficacy in Pancreatic Cancer. ACS Appl. Mater. Interfaces.

[168] Raza F., Evans L., Motallebi M., Zafar H., Pereira-Silva M., Saleem K., Peixoto D., Rahdar A., Sharifi E., Veiga F. (2022). Liposome-based diagnostic and therapeutic applications for pancreatic cancer. Acta Biomater..

[169] Wang X., Liu Y., Xu W., Jia L., Chi D., Yu J., Wang J., He Z., Liu X., Wang Y. (2021). Irinotecan and berberine co-delivery liposomes showed improved efficacy and reduced intestinal toxicity compared with Onivyde for pancreatic cancer. Drug Deliv. Transl. Res..

[170] Ranjan A.P., Mukerjee A., Helson L., Gupta R., Vishwanatha J.K. (2013). Efficacy of liposomal curcumin in a human pancreatic tumor xenograft model: Inhibition of tumor growth and angiogenesis. Anticancer. Res..

[171] Zinger A., Koren L., Adir O., Poley M., Alyan M., Yaari Z., Noor N., Krinsky N., Simon A., Gibori H. (2019). Collagenase Nanoparticles Enhance the Penetration of Drugs into Pancreatic Tumors. ACS Nano.

[172] Wang Y., Gao F., Jiang X., Zhao X., Wang Y., Kuai Q., Nie G., He M., Pan Y., Shi W. (2019). Co-Delivery of Gemcitabine and Mcl-1 SiRNA via Cationic Liposome-Based System Enhances the Efficacy of Chemotherapy in Pancreatic Cancer. J. Biomed. Nanotechnol..

[173] Passero F.C., Grapsa D., Syrigos K.N., Saif M.W. (2016). The safety and efficacy of Onivyde (irinotecan liposome injection) for the treatment of metastatic pancreatic cancer following gemcitabine-based therapy. Expert Rev. Anticancer. Ther..

[174] Wang-Gillam A., Li C.-P., Bodoky G., Dean A., Shan Y.-S., Jameson G., Macarulla T., Lee K.-H., Cunningham D., Blanc J.F. (2016). Nanoliposomal irinotecan with fluorouracil and folinic acid in metastatic pancreatic cancer after previous gemcitabine-based therapy (NAPOLI-1): A global, randomised, open-label, phase 3 trial. Lancet.

[175] Kaida S., Cabral H., Kumagai M., Kishimura A., Terada Y., Sekino M., Aoki I., Nishiyama N., Tani T., Kataoka K. (2010). Visible Drug Delivery by Supramolecular Nanocarriers Directing to Single-Platformed Diagnosis and Therapy of Pancreatic Tumor ModelVisible DDS for Diagnosis and Therapy of Solid Tumors. Cancer Res..

[176] Singh A.P., Biswas A., Shukla A., Maiti P. (2019). Targeted therapy in chronic diseases using nanomaterial-based drug delivery vehicles. Signal Transduct. Target. Ther..

[177] Srivastava A., Yadav T., Sharma S., Nayak A., Kumari A.A., Mishra N. (2015). Polymers in drug delivery. J. Biosci. Med..

[178] Wang G., Zhou Z., Zhao Z., Li Q., Wu Y., Yan S., Shen Y., Huang P. (2020). Enzyme-Triggered Transcytosis of Dendrimer–Drug Conjugate for Deep Penetration into Pancreatic Tumors. ACS Nano.

[179] Wu S.-T., Fowler A., Garmon C.B., Fessler A.B., Ogle J.D., Grover K.R., Allen B.C., Williams C.D., Zhou R., Yazdanifar M. (2018). Treatment of pancreatic ductal adenocarcinoma with tumor antigen specific-targeted delivery of paclitaxel loaded PLGA nanoparticles. BMC Cancer.

[180] Sun J., Wan Z., Chen Y., Xu J., Luo Z., Parise R.A., Diao D., Ren P., Beumer J.H., Lu B. (2020). Triple drugs co-delivered by a small gemcitabine-based carrier for pancreatic cancer immunochemotherapy. Acta Biomater..

[181] Sun I.-C., Yoon H.Y., Lim D.-K., Kim K. (2020). Recent Trends in In Situ Enzyme-Activatable Prodrugs for Targeted Cancer Therapy. Bioconjug. Chem..

[182] Santoni M., Miccini F., Cimadamore A., Piva F., Massari F., Cheng L., Lopez-Beltran A., Montironi R., Battelli N. (2021). An update on investigational therapies that target STAT3 for the treatment of cancer. Expert Opin. Investig. Drugs.

[183] Bimonte S., Barbieri A., Leongito M., Piccirillo M., Giudice A., Pivonello C., de Angelis C., Granata V., Palaia R., Izzo F. (2016). Curcumin AntiCancer Studies in Pancreatic Cancer. Nutrients.

[184] Bagley A.F., Ludmir E.B., Maitra A., Minsky B.D., Smith G.L., Das P., Koong A.C., Holliday E.B., Taniguchi C.M., Katz M.H. (2022). NBTXR3, a first-in-class radioenhancer for pancreatic ductal adenocarcinoma: Report of first patient experience. Clin. Transl. Radiat. Oncol..

[185] Bort G., Lux F., Dufort S., Crémillieux Y., Verry C., Tillement O. (2020). EPR-mediated tumor targeting using ultrasmall-hybrid nanoparticles: From animal to human with theranostic AGuIX nanoparticles. Theranostics.

[186] Li L., Song Y., He J., Zhang M., Liu J., Ni P. (2019). Zwitterionic shielded polymeric prodrug with folate-targeting and pH responsiveness for drug delivery. J. Mater. Chem. B.

[187] Vrettos E.I., Mező G., Tzakos A.G. (2018). On the design principles of peptide–drug conjugates for targeted drug delivery to the malignant tumor site. Beilstein J. Org. Chem..

[188] Wang Y., Cheetham A.G., Angacian G., Su H., Xie L., Cui H. (2017). Peptide–drug conjugates as effective prodrug strategies for targeted delivery. Adv. Drug Deliv. Rev..

[189] Alas M., Saghaeidehkordi A., Kaur K. (2020). Peptide–Drug Conjugates with Different Linkers for Cancer Therapy. J. Med. Chem..

[190] Chavda V.P., Solanki H.K., Davidson M., Apostolopoulos V., Bojarska J. (2022). Peptide-Drug Conjugates: A New Hope for Cancer Management. Molecules.

[191] Guo X., Wang L., Wei X., Zhou S. (2016). Polymer-based drug delivery systems for cancer treatment. J. Polym. Sci. Part A Polym. Chem..

[192] Manzur A., Oluwasanmi A., Moss D., Curtis A., Hoskins C. (2017). Nanotechnologies in Pancreatic Cancer Therapy. Pharmaceutics.

[193] Seifu M.F., Nath L.K. (2019). Polymer-Drug Conjugates: Novel Carriers for Cancer Chemotherapy. Polym. Technol. Mater..

[194] Mosiane K.S., Nweke E.E., Balogun M., Fru P.N. (2023). Polyethyleneglycol-Betulinic Acid (PEG-BA) Polymer-Drug Conjugate Induces Apoptosis and Antioxidation in a Biological Model of Pancreatic Cancer. Polymers.

[195] Almawash S.A., Mondal G., Mahato R.I. (2018). Coadministration of Polymeric Conjugates of Docetaxel and Cyclopamine Synergistically Inhibits Orthotopic Pancreatic Cancer Growth and Metastasis. Pharm. Res..

[196] Arias-Pinilla G.A., Modjtahedi H. (2021). Therapeutic Application of Monoclonal Antibodies in Pancreatic Cancer: Advances, Challenges and Future Opportunities. Cancers.

[197] Tolcher A.W. (2016). Antibody drug conjugates: Lessons from 20 years of clinical experience. Ann. Oncol..

[198] Birrer M.J., Moore K.N., Betella I., Bates R.C. (2019). Antibody-Drug Conjugate-Based Therapeutics: State of the Science. JNCI J. Natl. Cancer Inst..

[199] Parslow A.C., Parakh S., Lee F.-T., Gan H.K., Scott A.M. (2020). Antibody–drug conjugates for cancer therapy. Molecules.

[200] Sorbara M., Cordelier P., Bery N. (2022). Antibody-Based Approaches to Target Pancreatic Tumours. Antibodies.

[201] Nagaoka K., Bai X., Ogawa K., Dong X., Zhang S., Zhou Y., Carlson R.I., Jiang Z.-G., Fuller S., Lebowitz M.S. (2019). Anti-tumor activity of antibody drug conjugate targeting aspartate-β-hydroxylase in pancreatic ductal adenocarcinoma. Cancer Lett..

[202] Nishigaki T., Takahashi T., Serada S., Fujimoto M., Ohkawara T., Hara H., Sugase T., Otsuru T., Saito Y., Tsujii S. (2020). Anti-glypican-1 antibody–drug conjugate is a potential therapy against pancreatic cancer. Br. J. Cancer.

[203] Huang J., Agoston A.T., Guo P., Moses M.A. (2020). A Rationally Designed ICAM1 Antibody Drug Conjugate for Pancreatic Cancer. Adv. Sci..

[204] Xu J., Li X., Du Y. (2022). Antibody–Pattern Recognition Receptor Agonist Conjugates: A Promising Therapeutic Strategy for Cancer. Adv. Biol..

[205] Li W., Guo H., Li L., Zhang Y., Cui J. (2021). The promising role of antibody drug conjugate in cancer therapy: Combining targeting ability with cytotoxicity effectively. Cancer Med..

[206] Drago J.Z., Modi S., Chandarlapaty S. (2021). Unlocking the potential of antibody–drug conjugates for cancer therapy. Nat. Rev. Clin. Oncol..

[207] Marei H.E., Cenciarelli C., Hasan A. (2022). Potential of antibody–drug conjugates (ADCs) for cancer therapy. Cancer Cell Int..

[208] Lindberg J., Nilvebrant J., Nygren P., Lehmann F. (2021). Progress and Future Directions with Peptide-Drug Conjugates for Targeted Cancer Therapy. Molecules.

[209] Xu L., Xu S., Xiang T., Liu H., Chen L., Jiang B., Yao J., Zhu H., Hu R., Chen Z. (2022). Multifunctional building elements for the construction of peptide drug conjugates. Eng. Regen..

[210] Hoppenz P., Els-Heindl S., Beck-Sickinger A.G. (2020). Peptide-Drug Conjugates and Their Targets in Advanced Cancer Therapies. Front. Chem..

[211] Heh E., Allen J., Ramirez F., Lovasz D., Fernandez L., Hogg T., Riva H., Holland N., Chacon J. (2023). Peptide Drug Conjugates and Their Role in Cancer Therapy. Int. J. Mol. Sci..

[212] Cooper B.M., Iegre J., Donovan D.H.O., Halvarsson M., Spring D.R. (2021). Peptides as a platform for targeted therapeutics for cancer: Peptide–drug conjugates (PDCs). Chem. Soc. Rev..

[213] Berillo D., Yeskendir A., Zharkinbekov Z., Raziyeva K., Saparov A. (2021). Peptide-Based Drug Delivery Systems. Medicina.

[214] Fu C., Yu L., Miao Y., Liu X., Yu Z., Wei M. (2022). Peptide–drug conjugates (PDCs): A novel trend of research and development on targeted therapy, hype or hope?. Acta Pharm. Sin. B.

[215] Moore K.M., Desai A., Delgado B.D.L., Trabulo S.M.D., Reader C., Brown N.F., Murray E.R., Brentnall A., Howard P., Masterson L. (2020). Integrin αvβ6-specific therapy for pancreatic cancer developed from foot-and-mouth-disease virus. Theranostics.

[216] Worm D.J., Els-Heindl S., Beck-Sickinger A.G. (2020). Targeting of peptide-binding receptors on cancer cells with peptide-drug conjugates. Pept. Sci..

[217] de Mendoza T.H., Mose E.S., Botta G.P., Braun G.B., Kotamraju V.R., French R.P., Suzuki K., Miyamura N., Teesalu T., Ruoslahti E. (2021). Tumor-penetrating therapy for β5 integrin-rich pancreas cancer. Nat. Commun..

[218] Peng Z.-H., Kopeček J. (2015). Enhancing Accumulation and Penetration of HPMA Copolymer–Doxorubicin Conjugates in 2D and 3D Prostate Cancer Cells via iRGD Conjugation with an MMP-2 Cleavable Spacer. J. Am. Chem. Soc..

[219] Kang S., Lee S., Park S. (2020). iRGD Peptide as a Tumor-Penetrating Enhancer for Tumor-Targeted Drug Delivery. Polymers.

[220] Peng Z.-H., Jogdeo C.M., Li J., Xie Y., Wang Y., Sheinin Y.M., Kopeček J., Oupický D. (2022). Tumor Microenvironment-Responsive Polymeric iRGD and Doxorubicin Conjugates Reduce Spontaneous Lung Metastasis in an Orthotopic Breast Cancer Model. Pharmaceutics.

[221] Ragozin E., Hesin A., Bazylevich A., Tuchinsky H., Bovina A., Zahavi T.S., Oron-Herman M., Kostenich G., Firer M., Rubinek T. (2018). New somatostatin-drug conjugates for effective targeting pancreatic cancer. Bioorg. Med. Chem..

[222] Dean A., Gill S., McGregor M., Broadbridge V., Järveläinen H.A., Price T. (2022). Dual αV-integrin and neuropilin-1 targeting peptide CEND-1 plus nab-paclitaxel and gemcitabine for the treatment of metastatic pancreatic ductal adenocarcinoma: A first-in-human, open-label, multicentre, phase 1 study. Lancet Gastroenterol. Hepatol..

[223] Von Hoff D.D., Ervin T., Arena F.P., Chiorean E.G., Infante J., Moore M., Seay T., Tjulandin S.A., Ma W.W., Saleh M.N. (2013). Increased Survival in Pancreatic Cancer with nab-Paclitaxel plus Gemcitabine. N. Engl. J. Med..

[224] Chen B., Dai W., He B., Zhang H., Wang X., Wang Y., Zhang Q. (2017). Current Multistage Drug Delivery Systems Based on the Tumor Microenvironment. Theranostics.

[225] Blanco E., Hsiao A., Mann A.P., Landry M.G., Meric-Bernstam F., Ferrari M. (2011). Nanomedicine in cancer therapy: Innovative trends and prospects. Cancer Sci..

[226] Blanco E., Sangai T., Hsiao A., Ferrati S., Bai L., Liu X., Meric-Bernstam F., Ferrari M. (2013). Multistage delivery of chemotherapeutic nanoparticles for breast cancer treatment. Cancer Lett..

[227] Stylianopoulos T., Jain R.K. (2015). Design considerations for nanotherapeutics in oncology. Nanomed. Nanotechnol. Biol. Med..

[228] Martinez J., Brown B.S., Quattrocchi N., Evangelopoulos M., Ferrari M., Tasciotti E. (2012). Multifunctional to multistage delivery systems: The evolution of nanoparticles for biomedical applications. Chin. Sci. Bull..

[229] Stylianopoulos T., Wong C., Bawendi M.G., Jain R.K., Fukumura D. (2012). Multistage nanoparticles for improved delivery into tumor tissue. Methods in Enzymology.

[230] Wong C., Stylianopoulos T., Cui J., Martin J., Chauhan V.P., Jiang W., Popović Z., Jain R.K., Bawendi M.G., Fukumura D. (2011). Multistage nanoparticle delivery system for deep penetration into tumor tissue. Proc. Natl. Acad. Sci. USA.

[231] Liang T., Yao Z., Ding J., Min Q., Jiang L.-P., Zhu J.-J. (2018). Cascaded Aptamers-Governed Multistage Drug-Delivery System Based on Biodegradable Envelope-Type Nanovehicle for Targeted Therapy of HER2-Overexpressing Breast Cancer. ACS Appl. Mater. Interfaces.

[232] Yu Y., Zhang X., Qiu L. (2014). The anti-tumor efficacy of curcumin when delivered by size/charge-changing multistage polymeric micelles based on amphiphilic poly(β-amino ester) derivates. Biomaterials.

[233] Serda R.E., Godin B., Blanco E., Chiappini C., Ferrari M. (2011). Multi-stage delivery nano-particle systems for therapeutic applications. Biochim. Et Biophys. Acta (BBA)-Gen. Subj..

[234] Cun X., Chen J., Li M., He X., Tang X., Guo R., Deng M., Li M., Zhang Z., He Q. (2019). Tumor-Associated Fibroblast-Targeted Regulation and Deep Tumor Delivery of Chemotherapeutic Drugs with a Multifunctional Size-Switchable Nanoparticle. ACS Appl. Mater. Interfaces.

[235] Li H.J., Du J.Z., Liu J., Du X.J., Shen S., Zhu Y.H., Wang X., Ye X., Nie S., Wang J. (2016). Smart superstructures with ultrahigh pH-sensitivity for targeting acidic tumor microenvironment: Instantaneous size switching and improved tumor penetration. ACS Nano.

